# Constrained and Open Mesoporosity in Polypropylene Cracking: Insight From Spectroscopic Investigations of Acidity, Diffusion, and Activity

**DOI:** 10.1021/acs.langmuir.3c03880

**Published:** 2024-03-23

**Authors:** Karolina A. Tarach, Gabriela Jajko, Miguel Palomino, Fernando Rey, Kinga Góra-Marek

**Affiliations:** † Faculty of Chemistry, Jagiellonian University in Kraków, Gronostajowa 2, Kraków 30-387, Poland; ‡ Doctoral School of Exact and Natural Sciences, Jagiellonian University in Krakow, Łojasiewicza 11, Krakow 30-348, Poland; § Instituto de Tecnología Química, Universitat Politècnica de València − Consejo Superior de Investigaciones Científicas (UPV-CSIC), Avda. de los Naranjos s/n, Valencia 46022, Spain

## Abstract

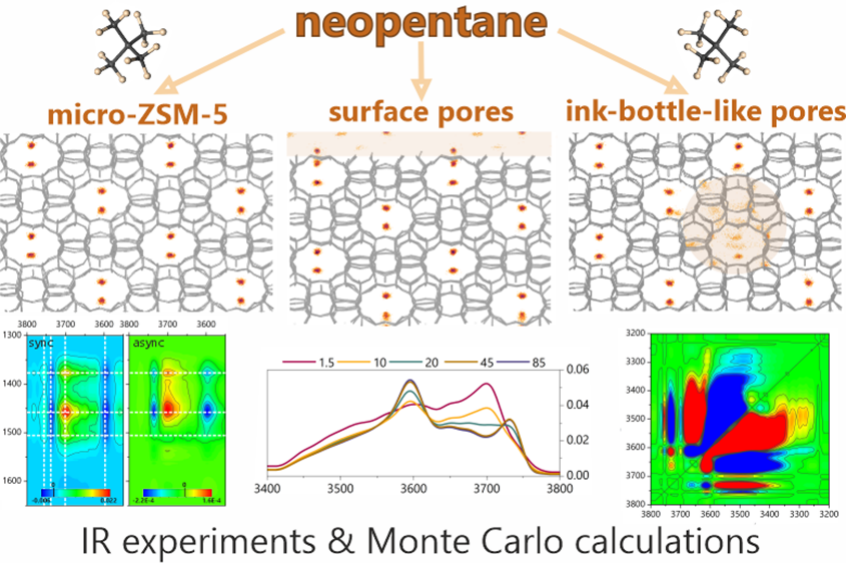

The outcome of the demetalation process of zeolites depends on applied treatment conditions and can lead to the formation of either open or constrained mesopores. The quaternary ammonium cations as pore-directing agents during desilication are responsible for developing constrained mesoporosity with bottleneck entrances. However, higher mesopore surface area and higher accessibility of acid sites are often found for the hierarchical zeolites with constrained mesopores. This is followed by better catalytic activity in the cracking of vacuum gas oil and polymers. For desilication with pure NaOH, a realumination process is observed and an additional acid-wash step is required to reach their full catalytic potential. Thus, this study aims to analyze the acidic and catalytic properties of hierarchical ZSM-5 zeolites of different mesoporosity types employing *in situ* and *operando* FT-IR spectroscopic evaluation of polypropylene cracking. The suitability of constrained mesoporosity is studied by assessing the neopentane diffusion in kinetic adsorption, Monte Carlo calculations, and rapid scan FT-IR spectroscopic measurement analyzed by Crank solution for diffusion. The FT-IR spectroscopic results of *in situ* and *operando* studies are supported by two-dimensional correlation analysis, allowing to establish the direction of changes seen on spectra and their order.

## Introduction

The applicability of hierarchical zeolites in reactions involving the cracking of polymers or gas oil is of great interest as the high activity and selectivity of these catalysts are found.^1−3^ Furthermore, the hierarchical catalysts showed some similarities between the cracking of vacuum gas oil and polymers in the manner of the activity and selectivity.^4^ The development of a secondary pore system reflects not only in porosity but also in the acidic picture of modified catalysts.^5^ Demetalation processes appear as highly versatile methods of hierarchical zeolites preparation, independently from the Si/Al ratio, 10- or 12-ring structure type, or grains size the proper usage of desilication/dealumination will allow for development of secondary porosity.^6,7^ Advantageous shortening of the diffusion path and improved accessibility of acid sites for bulky reagents might ensure, among others, limited coking.^8^ However, those alternations in zeolite structure promote the formation of extraframework Lewis acid sites in large amounts.^9−11^ Thus, the full catalytic activity of hierarchical zeolites usually can be reached only by properly adjusted conditions of a series of modification treatments.^6^ The desilication process can lead to the formation of open or constrained mesopores^3,12^ depending on the applied desilicating agent. Using quaternary ammonium cations as pore directing agents (PDA) during desilication leads to constrained mesopores with bottleneck entrances but bigger mesopores surface thus higher accessibility of acid sites.^13,14^ The open mesopores offered higher catalytic lifetime in the methanol-to-hydrocarbon process^12^ and activity in the cracking of vacuum gas oil;^15^ however, when compared with the catalysts of constrained mesoporosity, the latter often showed higher catalytic activity in the cracking of vacuum gas oil and/or polymers without severe coking.^1,3,16^

The other consequence of the desilication process is the formation of Lewis acid sites occurring in the realumination process and located in the majority on the surface of mesopores. Their appearance is attributed to the facile dehydroxylation of nonframework protonic sites of the Al-rich shell, which was proved during in situ FT-IR spectroscopic studies of the calcination of freshly desilicated ZSM-5.^10,17^ With increasing the calcination temperature, the protonic sites originating from the Al-rich shell are transformed to Lewis acid sites typical of those coming from dehydroxylation. Furthermore, the full catalytic potential in cracking reactions involving the desilicated zeolites with open mesopores was achieved after removing the Al-rich shell from the outer part of the zeolite grains.^6,11^ However, this must be verified for zeolites with constrained mesoporosity obtained in PDA desilication.

This study aims to assess the influence of the PDA presence during the desilication process on specific properties of derived hierarchical zeolites. The analysis of acidic and catalytic properties supported by studies of diffusion of neopentane, both experimental and theoretical, based on Monte Carlo calculations, will be provided. The advanced *in situ* and *operando* FT-IR spectroscopy technique supported by chromatographic and mass spectrometry methods were employed to evaluate the acidity, diffusivity, and catalytic activity of studied catalysts. To provide significant insight into the processes occurring during the diffusion of neopentane and catalytic cracking of polymers, the FT-IR spectroscopic data were also assessed by two-dimensional correlation analysis (2D CoS) to establish the direction of changes and their order.

The 2D CoS analysis^18,19^ enhances the coupling between the bands identified at given frequencies on synchronous maps; either two bands change in the same or opposite direction, depending on the positive or negative sign of the peak. The asynchronous maps with peaks at the matching positions deliver the order of the coupled changes for the bands. If the signs of peaks on both maps are identical, then the band of higher frequency changes first; if the signs are negative, then the band located at lower frequency changes before the other one. The *in situ* and *operando* FT-IR spectroscopic experiments gained significant insight from the 2D CoS analysis.

## Experimental Section

### Catalyst Preparation

Two hierarchical zeolites were derived from a common ZSM-5 (NH_4_-form, CBV 5524G, SiAl = 32, Zeolyst International) by leaching it with 0.2 M NaOH or 0.2 M NaOH&TBAOH (60:40) solutions at 80 °C for half an hour.^6^ The zeolite treated with NaOH is denoted with deSi, while the one desilicated in a mixture of NaO&TBAOH is denoted with deSi&PDA; the TBAOH serves as pore directing agent.^20^ Typically, 100 mL of the alkaline solutions was added to 3.0 g of zeolite. After each treatment, the suspension was cooled in an ice bath and filtered. The resulting hierarchical zeolites were washed with deionized until neutral pH, ion-exchanged three times with a 0.5 M NH_4_NO_3_ solution at 60 °C for 1 h, then filtered, washed, and dried at 100 °C for 24 h. After desilication, the dealumination was performed at a temperature of 80 °C for 1 h using a 0.05 M solution of HNO_3_. After the acid treatment, the samples were filtered, washed, and dried at 100 °C for 24 h. All modified samples were calcined at 550 °C for 5 h at a 2 °C/min rate. The parent material in protonic form after calcination was denoted as micro-ZSM-5.

### Standard Experimental Methods for Structural, Textural, and Acidity Characterization

The powder X-ray diffraction (XRD) patterns were gathered from a Rigaku Multiflex diffractometer (Cu Kα, 40 kV, 40 mA). The relative crystallinity (Rel. Cryst., %) was evaluated by comparing the integrated area of the characteristic reflections of the MFI type structure in the range between 22.5 and 25.0°. The micro-ZSM-5 sample was taken as a referenced value.

The Si and Al content was derived from inductively coupled plasma–optical emission spectroscopy (ICP-OES, PerkinElmer, Optima 2100DV). Typically, 50–80 mg of sample was digested in a Teflon vessel using HCl (35%) and HF (48%) in a ratio of 10:1. The final concentration was adjusted with deionized water.

The low-temperature sorption of nitrogen measurements were realized on a Quantachrome Autosorb-1-MP gas sorption analyzer. The pretreatment of samples included heating to 350 °C and evacuation under high vacuum conditions (10^–5^ mbar) for 16 h. The micropore volume (*V*_micro_) and surface area of the micropores (*S*_micro_) were calculated by the *t*-plot method. The specific surface area (*S*_BET_) was calculated based on the Brunauer–Emmet–Teller (BET) method, following the Rouquerol et al. recommendations.^21^ The pore size distributions were obtained from the adsorption isotherm branch using the Barrett–Joyner–Halenda (BJH) model.^22^ The *S*_meso_ was calculated as a difference between *S*_BET_ and *S*_micro_.

The FT-IR studies using probe molecules for acidity and accessibility assessment were performed on a Vertex 70 spectrometer (Bruker) equipped with an MCT detector with a resolution of 2 cm^–1^ and a scanner velocity of 20 kHz. The spectra for quantitative analysis were normalized to the same sample mass (10 mg). The study was performed with the use of pyridine (Py, ≥99.8%, Sigma-Aldrich), pivalonitrile (Pn, 98%, Sigma-Aldrich), and carbon monoxide (CO, Linde Gas Poland, 99.95%) as probe molecules. Before the analysis, the samples were pressed as self-supporting discs (5–10 mg·cm^–2^) and pretreated *in situ* at 520 °C under high-vacuum (10^–6^ mbar) conditions for 1 h.

For acid site concentration assessment, the excess of Py (>20 Tr) was sorbed sufficiently to neutralize all acid sites at 170 °C. Then, the gaseous and physisorbed molecules were removed under high vacuum conditions at the same temperature. The acid sites concentration was assessed based on the respective bands’ intensities (band height) and their absorption coefficients (1545 cm^–1^, PyH^+^, 0.07 cm^2^·μmol^–1^ and the 1450 cm^–1^, PyL, 0.10 cm^2^·μmol^–1^).^23^ The acid strength was determined by thermodesorption experiments. The preservation of the 1545 (PyH^+^) and 1455 cm^–1^ (PyL) bands upon desorption at 450 °C was taken. Then, the ratios of PyH^+^_450_/ PyH^+^_170_ and PyL_450_/ PyL_170_ were used to calculate the acid strength of the Bro̷nsted and Lewis sites, respectively.

For accessibility of acid sites, the Pn sorption in an excess amount was realized at room temperature. Then, the gaseous and physisorbed molecules were removed under high vacuum conditions at the same temperature. The concentration of the sites accessible to the Pn molecules was estimated based on respective band intensities (band height) and their absorption coefficients (2277 cm^–1^, Pn···H^+^, 0.11 cm^2^·μmol^–1^ and 2305 cm^–1^, Pn···L, 0.15 cm^2^·μmol^–1^).^13^

The sorption of CO was performed at −100 °C. The dosing of CO was performed until total saturation of Bro̷nsted acid sites was observed. The downshift (Δν_CO···OH_) of the Si(OH)Al···CO band of the adducts was taken as a measure of the acid strength.

### FT-IR Spectroscopic Studies of Neopentane Sorption

For studies of neopentane diffusion over zeolites, the samples were pressed into self-supporting pellets and placed in a homemade quartz IR cell. The samples were pretreated at 520 °C under high vacuum (10^–5^ Pa) and then cooled to the temperature of measurement (25 °C). The spectra were recorded on a Vertex 70 (Bruker) spectrometer equipped with an MCT detector; the resolution was 2 cm^–1^. The rapid scan (RS) measurements of neopentane diffusion ability were realized with the scanning velocity increased to 80 kHz. Thus, the accumulation of one spectrum was taken within 0.3 s. A total of 1500 spectra were acquired in a single measurement. The absolute pressure applied during a single measurement was 520 Pa. The diffusion ability of the studied zeolite was measured in terms of its characteristic diffusion time constant (*D*/*r*^2^) values. In this parameter, *D* is Fick’s diffusion coefficient, and *r* is the averaged radius representative of the sample crystal size (400 nm), assuming the spherical particles. Therefore, the diffusion rate constant *D*/*r*^2^ [s^–1^], independent from the crystal size, can be derived from adsorption kinetic measurements by using the first ten terms of the Crank solution for diffusion:^24^
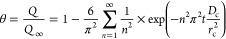
1where *Q* represents the neopentane uptake at a time *t* and *Q*_∞_ is the uptake at the equilibrium. The uptake for FT-IR spectroscopic studies is the integration of C–H stretching bands in the region of 3100–2700 cm^–1^. The detailed procedure of calculations and comparison for other hydrocarbons is presented in our previous paper.^25^

### Kinetic Neopentane Sorption Measurements

Adsorption kinetics of neopentane were measured at 25 °C in a Bel Max II volumetric analyzer from Microtrac MRB. The instrument was customized with a larger expansion volume attached to the manifold to dose the gas while maintaining a pressure variation below 10%. Approximately 50 mg of sample were placed and carefully weighted in a glass tube. Before each adsorption experiment, the sample was outgassed at 325 °C under a final pressure of 10^–5^ Pa for at least 8 h in an external unit. The sample was then cooled under a high vacuum down to room temperature, and the tube was filled with nitrogen to calculate the dry amount of the sample. The sample tube was then mounted in the Bel Max II analyzer and degassed again at 325 °C under a high turbomolecular vacuum overnight. The temperature was finally decreased to 25 °C using a recirculating thermostatic bath. Adsorption kinetics were then performed by introducing neopentane in the manifold up to the desired pressures and then expanding it to the glass tube containing the degassed sample by acquisition of the adsorbed amount of gas as a function of time. Diffusion rate constants *D*/*r*^2^ [s^–1^] of neopentane at 25 °C were calculated based on collected data on a BEL MAX II sorption analyzer and fitted automatically with BEL-Master software according to the Crank theory.^24^ The data is presented as the  vs time, where *C* is the density from ideal gas state equation *p* = *CRT* and *C*_0_ and *C*_en_ are the initial and at the *n*th point density, respectively.^26^

### Calculations for Adsorption and Diffusion of Neopentane

Grand-canonical Monte Carlo (GCMC) simulations were used to compute the adsorption isotherms of neopentane. Each point on the adsorption isotherm was computed by running 5 × 10^4^ initialization cycles and 5 × 10^6^ production cycles of translation, rotation, swap, and reinsertion, with equal probabilities. The Peng–Robinson equation of state^27^ was used to relate the pressures and fugacity of the pure components. The Lennard–Jones potentials were truncated and shifted at a cutoff distance of 12 Å. For each zeolite, computational models were designed with a Si/Al ratio close to the experiments: Si/Al = 31 for micro-ZSM-5, Si/Al = 24 for deSi&PDA-ZSM-5, and Si/Al = 21 for deSi-ZSM-5. For deSi&PDA-ZSM-5, the structure was designed with the bottle-like pore in the *YZ* direction, and the diameter of the bottleneck/entrance to the pore was approximately 7 Å. For deSi-ZSM-5, the biggest issue was the location of neopentane particles in the slab space. For this reason, it was essential to adjust its size so that particles would not accumulate there, so the slab size was set to 3 Å. All calculations were performed in a 2 × 2 × 2 unit cell simulation box with applied periodic boundary conditions,^28^ using RASPA code.^29^ Additional calculation details are provided in the Supporting Information (eqs S1–S6).

The force field parameters for the host structure were taken from the GenericZeolites force field provided by RASPA. To describe the neopentane molecule, we used the TraPPE force field^30^ (Table S1). Interactions with oxygen atoms were appropriately modified (Table S2) immediately after application of the mixing rules.

Self-diffusion coefficients were calculated using Molecular Dynamics (MD) simulations, where the following system configurations are generated by integrating Newton’s laws of motion. This leads to a trajectory describing the particles’ positions, velocities, and accelerations as they change over time. Calculations were performed by using the NVT ensemble at the temperature corresponding to the experiments. Self-diffusion coefficients were calculated by taking the slope of the root-mean-square displacement over extended times.^31^

### Thermogravimetric Analysis of Polypropylene Catalytic Cracking

The catalytic and thermal cracking was assessed by thermogravimetric analysis using a TGA/SDTA Mettler Toledo apparatus. For catalytic cracking, the catalyst powder (10 mg) and polypropylene (30 mg) were mixed (10 min) in an agate mortar. The polypropylene (PP, PP, Sigma-Aldrich, product no.: 428116, lot no.: MKCH4322, *M*_w_ of 12,000, *M*_n_ of 5000, density of 0.9 g/mL, average size of 0.4 mm) was not subjected to any modifications. Then, a portion of the mixture (ca. 10 mg) was pressed (2 atm) in the form of a disc (0.2 cm^2^), then transferred to α-Al_2_O_3_ crucible, and weighed with Mettler Toledo balance before the analysis. The decomposition of polypropylene was carried out in a temperature range from 30 to 600 °C at the heating rate of 5 °C min^–1^ under nitrogen flow (80 mL min^–1^). The conversion calculations considered the catalyst weight and the adsorbed moisture content. For thermal cracking, polypropylene was cracked without the addition of catalysts. The coke content was calculated from the TG experiments. After the polymer cracking, the sample in the nitrogen flow was cooled down to room temperature and then was subjected to coke burning off with a rate of 30 °C min^–1^ to 800 °C in a flow of synthetic air (80 mL min^–1^) until no mass change was observed.

### *Operando* FT-IR-GC-MS Studies of Polypropylene Cracking

The catalyst’s activity in PP cracking was also studied in an *operando* system connected to a flow setup with nitrogen as a carrier gas (30 mL/min). The zeolites were mixed with PP (1:1) and pressed into a self-supporting wafer (*ca.* 5.5–6 mg/cm^2^). The wafer was placed in a custom-made 2 cm^3^-volume IR quartz gas cell. MeasLine (www.measline.com) company delivered the customized spectroscopic cell under a licensed patent (PL232633, Poland).^32^ The reaction cell allows for the simultaneous analysis of the gas phase and surface of the catalysts during the reaction. The homogeneity of the zeolite/catalyst mixture used for TGA and *operando* purposes was followed by comparing their IR spectra collected at room temperature and normalized to the same intensity of the overtone bands (2050–1800 cm^–1^). Each catalyst was reflected in the same intensity of the bands 2960 (−CH_3_ group) and 2925 cm^–1^ (−CH_2_ group) and thus the equal proportion of PP and LDPE in all materials. The carrier gas was transferred by 1/16 in. Teflon lines, heat-traced at 110 °C. The *operando* IR cell with placed catalyst disc was rapidly heated from room temperature up to 250 °C with the ramping rate of 10 °C·s^–1^. Time-resolved FT-IR spectra were taken on a Vertex 70 Bruker FT-IR spectrometer (resolution = 2 cm^–1^) during 60 min of reaction. The catalysts wafer in the cell was rapidly heated from room temperature up to 250 °C at a 10 °C/s ramping rate, and in parallel to FT-IR spectroscopic observations, mass spectrometry (MeasLine, www.measline.com, RGA200) and gas chromatography (Agilent Technologies 7890B) simultaneously analyzed the reaction products. The resulting coke and tar were considered in the selectivity of the catalysts. After the polymer cracking, the sample in the nitrogen flow was cooled to 200 °C and then subjected to coke burning off in a synthetic air flow (80 mL min^–1^) with a heating rate of 10 °C min^–1^. The temperatures of coke burning off were increasing sequentially between 300–600 °C; each time, the temperature was increased by 50 °C and stabilized for 10 min, the total oxidation process took 90 min. The relative content of CO_2_ (*m*/*z* = 44) and H_2_O (*m*/*z* = 18) was assessed with the use of mass spectrometry (MeasLine, www.measline.com, RGA200).

## Results and Discussion

### Acidity, Accessibility, and Nature of Sites in Studied Zeolites

XRD analysis of studied samples proved the conservation of MFI structure properties after each modification step (Figure S1). A similar conclusion can be drawn from N_2_-physisorption studies providing *V*_micro_ values typical for ZSM-5 zeolites (Table 1). A minor drop of *V*_micro_ was found in the PDA-treated samples. This was not followed by the presence of an amorphous phase or a significant decrease of XRD peaks intensity and thus should not negatively affect the catalytic activity of PDA-derived materials. Upon modification with NaOH or NaOH&TBAOH, the mesoporosity of various types was developed. The NaOH-derived samples presented relatively open or cylindrical mesopores, while the samples obtained in NaOH&TBAOH treatment possess bottle-ink-shaped mesopores of various diameters. The highest value of secondary mesopore surface was found for material treated with NaOH&TBAOH. The modified samples exhibited different Si/Al ratios; the desilication process selectively removed silicon; thus, the lower ratios were found afterward. The acid treatment was aimed to remove mainly the aluminum debris; therefore, a partial restoration of Si/Al ratios was observed. The Si/Al ratio change reflected the acidity and accessibility of sites generated by aluminum.

**Table 1:** Structural and Textural Parameters of Studied Catalysts

		rel. cryst.	*S*_BET_	*S*_micro_^a^	*S*_meso_^c^	*V*_micro_^a^	*V*_meso_^b^
	Si/Al	%	m^2^·g^–1^	m^2^·g^–1^	m^2^·g^–1^	cm^3^·g^–1^	cm^3^·g^–1^
micro-ZSM-5	32	100	377	337	40	0.17	0.06
deSi-ZSM-5	18	88	517	371	146	0.16	0.35
deSi-ZSM-5&Ac	25	87	501	357	144	0.15	0.34
deSi&PDA-ZSM-5	21	86	467	246	221	0.11	0.37
deSi&PDA-ZSM-5&Ac	24	84	505	277	229	0.12	0.39

a *t*-Plot method with thickness between 4 and 9.2 Å, *P*/*P*° (0.05, 0.8).

b Determined by the BJH method based on the adsorption curve of the isotherm for the pore size range from 17 to 300 Å.

c Calculated as a difference between *S*_BET_ and *S*_micro_.

The desilication process with NaOH assures moderate accessibility of acid sites (Table 2). Only the NaOH&TBAOH-derived samples present significantly higher AF_B_ values. Also, for NaOH-derived samples, acid-washing improves the accessibility of sites; nonesuch behavior is noticed for NaOH&TBAOH-modified catalysts. The NaOH-modified catalysts possess an Al-rich shell formed during the realumination process occurring on the surface of newly created mesopores. Though the acid site accessibility increases upon desilication, the full potential of NaOH-derived hierarchical zeolite can be reached only by further acid treatment.^6,11^ The catalysts derived upon NaOH&TBAOH treatment usually present higher accessibility of acid sites.^1,3,14,16^ As shown in Table 2, the acid treatment does not influence the accessibility of acid sites, as it already exceeds 50%.

**Table 2:** Acidic Parameters of Studied Zeolites

	*B*^a^ μmol·g^–1^	*L*^a^ μmol·g^–1^	PyH^+^_450_/PyH^+^_170_^a^	PyL_450_/PyL_170_^a^	AF_B_^b^ %	Δν_CO···OH_^c^ cm^–1^
micro-ZSM-5	443	41	0.94	0.99	12	315
deSi-ZSM-5	630	180	0.84	0.91	19	306
deSi-ZSM-5&Ac	484	98	0.84	0.98	36	303
deSi&PDA-ZSM-5	540	160	0.75	1.00	57	308
deSi&PDA-ZSM-5&Ac	470	110	0.78	0.87	56	311

a Data derived from Py adsorption IR studies: the concentration of Bro̷nsted (B), Lewis (L) acid sites, and the acid strength of sites (Py_450_/Py_170_).

b Accessibility of acid sites derived from Pn adsorption IR studies.

c Strength of the Si(OH)Al groups determined from low-temperature CO sorption IR experiments.

The Lewis acid sites were characterized in depth to understand the Al-rich shell’s nature better. The carbon monoxide is a probe molecule particularly devoted to discriminating between the Lewis sites of various properties. Such Lewis acid sites can be part of extra-framework aluminum species. As can be noticed (Figure S2), both deSi-ZSM-5 and deSi&PDA-ZSM-5 catalysts present a high amount of Lewis acid sites identified by the band at 2230–2226 cm^–1^. The acid treatment diminishes the content of the strong Lewis acid sites. However, the Bro̷nsted acid site accessibility is affected after the dealumination process for only NaOH-derived samples. Thus, it is anticipated that the location of strong Lewis acid sites from dehydroxylation differs depending on the desilicating agent, and only for NaOH-treated samples does it block the pore mouth entrances. Moreover, the heterogeneity of Lewis acid sites can be noticed for the deSi-ZSM-5 sample as also the Lewis acid sites identified by the band at 2188 cm^–1^. The quantitative evaluation of the total number of Lewis sites needs to be, however, based on pyridine sorption results. The observed discrepancy between the number of Lewis sites detected with Py and CO results from the various basicities of the probes. Some Lewis acid sites located in the acid-leached desilicated samples are not able to bind a weakly basic CO molecules in contrast to highly basic Py molecules (Table 2).

The studied catalyst offers differentiated textural and acidic properties; most notably, the accessibility of newly created mesopores and acid sites inside micropores differs strongly. Thus, the materials are anticipated to offer various diffusion and catalytic properties.

### Adsorption and Diffusion of Branched Neopentane in Microporous and Hierarchical Zeolites

The adsorption and diffusion processes of branched neopentane were followed in different approaches by rapid scan (RS) FT-IR spectroscopy, kinetic sorption experiments, and theoretical calculations. The RS FT-IR spectroscopic analysis results were further explored by two-dimensional correlation analysis (2D CoS). Neopentane adsorption on zeolites is dipole–induced dipole attraction (van der Waals interactions); thus, it can be followed as the perturbation of the Si(OH)Al groups with nonpolar neopentane.

**Figure 1:**
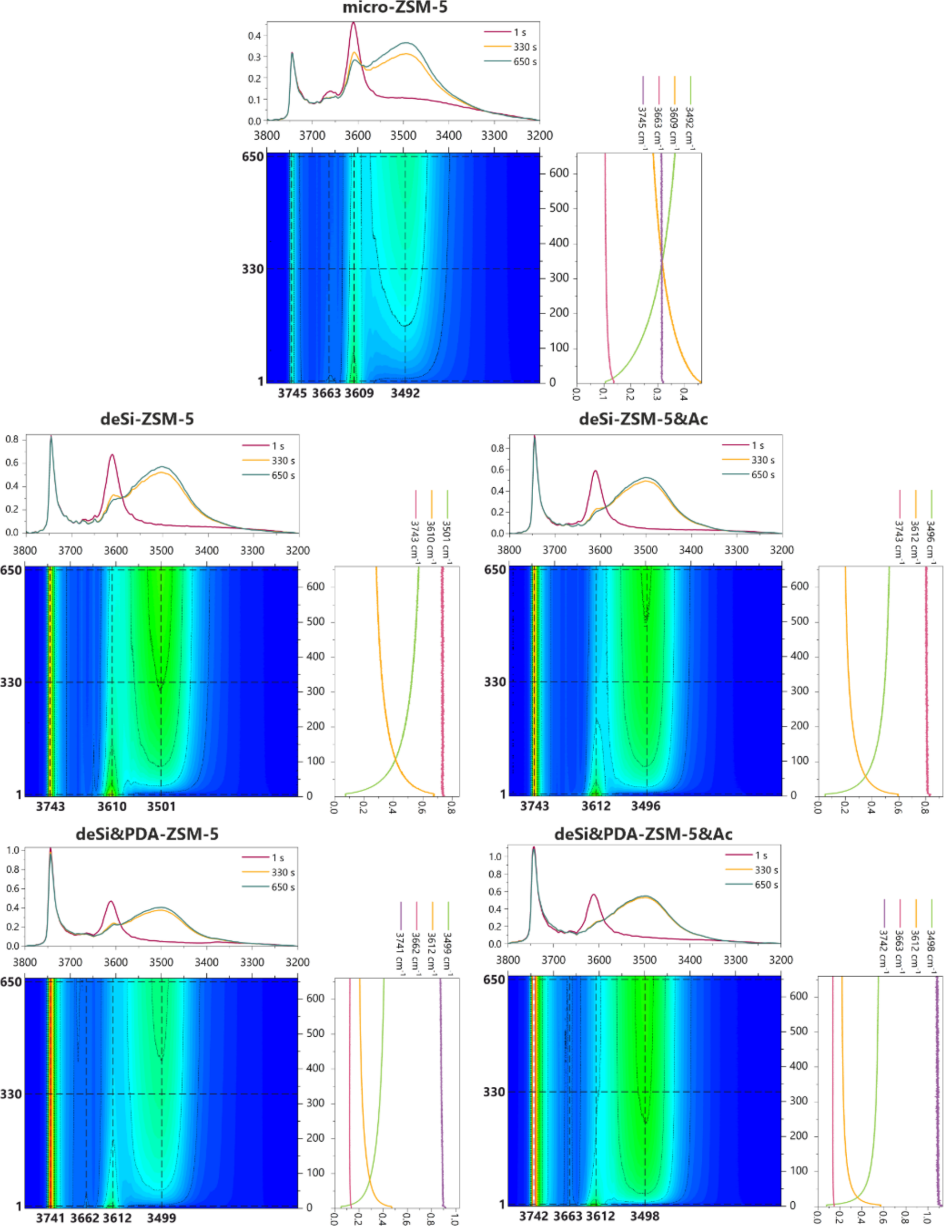
Top-down projection maps of RS FT-IR spectra in the stretching region of the O-H groups for all studied catalysts. Spectra collected during neopentane sorption (520 Pa, 60 °C, every 0.3 s during 660 s). Each upper panel over a 2D map contains spectra at a given time. The right panel shows the traces of selected wavenumbers changing with the time of neopentane sorption.

First, the analysis of the alternations of the O-H groups in zeolites upon neopentane sorption will be performed. The rapid scan time-resolved FT-IR spectra in the region of O–H stretching vibrations of the zeolites after the adsorption of neopentane at 60 °C and *p* = 520 Pa are presented in Figure 1. After the contact of zeolites with adsorbate molecules, the most pronounced changes are found for the band of the Si(OH)Al groups at ca. 3610 cm^–1^. The Si(OH)Al groups are engaged in bonding with neopentane molecules, as manifested by their decreased intensity. The modified samples are more prone to interaction with neopentane when compared to the parent sample. Among O–H groups involved in the interaction with neopentane, the Si(OH) and Al(OH) can also be found. However, this depends on the previous treatments of the sample and will be discussed in detail based on the results of 2D COS analysis. The comparison between the samples after 1, 330, and 650 s of neopentane introduction shows that in the parent sample, less than 50% of the Si(OH)Al band intensity decreases, while for modified samples, the drop is more significant. It becomes clear that the Si(OH)Al groups in the NaOH-derived samples offer lower susceptibility to interact with neopentane than those in NaOH&TBAOH-derived samples. The Si(OH)Al groups in the desi-PDA-ZSM-5&Ac sample after 650 s are fully engaged in bonding with neopentane.

2D CoS analysis of rapid scan FT-IR spectra in the region of the O–H group stretching bands during neopentane diffusion and sorption give insight into the order of events occurring on the zeolite surface (Figures S3 and S4). The most pronounced changes over the zeolite surface are related to diminishing the intensity of Si(OH)Al groups upon interaction with neopentane and increasing the intensity of a new broad band at ca. 3500 cm^–1^. However, a more detailed analysis of the O-H groups region on 2D CoS maps show that besides high similarities between the observed changes in the spectra (Figure S3), the order of events is different between the samples (Figure S4). The micro-ZSM-5 and NaOH-treated samples show the sequential order of changes: 3730, 3700, 3610, 3740, 3500, and 3660 cm^–1^. Meanwhile, for the deSi&PDA-treated sample, the order of changes in O–H bands was as follows: 3706, 3743, 3500, 3610, and 3650 cm^–1^. Thus, the Si(OH)Al groups are not the only ones involved in this interaction but also the silanols (3740, 3730, and 3700 cm^–1^) and the Al(OH) groups (3660–3650 cm^–1^). Furthermore, important differences in silanols interaction with neopentane between the studied zeolites are found. The silanols on the surface of micro-ZSM-5 and deSi-ZSM-5 samples are not involved in the interaction with the neopentane; only the subsequent acid treatment of the latter sample gives neopentane access to silanols in deSi-ZSM-5&Ac. In the case of both NaOH&TBAOH-treated samples, nontreated and acid-treated, the silanols are accessible for neopentane. The involvement of silanols seems crucial for the diffusion of neopentane because the deSi&PDA-treated sample of the external silanols (3740 cm^–1^) decreases their intensity upon interaction with neopentane before the Si(OH)Al groups. This implies that the treatment with NaOH gives a large external surface area rich in hydrogen-bonded silanols, most probably to the hydroxyl groups of adjacent silanol(s). Thus, those silanols create stable species and are not prone to interacting with neopentane.

Furthermore, the Al-rich shell and the Al(OH) groups in the NaOH-treated sample can significantly restrain the interaction between the silanols and neopentane. On the other hand, the silanols, which can interact with neopentane in NaOH&TBAOH treated samples, can serve as primary sorption centers during catalytic reactions and then increase the overall activity of the catalyst. The silanols in NaOH&TBAOH-derived interact with neopentane; thus, neopentane can reach micropore entrances faster than in samples with inert surfaces. Furthermore, the interaction of nonpolar neopentane molecule and surface species is ruled by the acidic property of the latter. The more acidic surface hydroxyls are, the higher is the strength of their interaction with the neopentane molecule. The stronger acidity of surface Si–OH groups than the Al–OH species results from higher electronegativity of silicon atom. As a result, some of the silanol groups have sufficient acidity for a series of catalytic processes in which strong acid sites, as Bronsted acid sites, are undesirable. Besides the acidic function, the Al-rich shell can importantly restrain the diffusion of neopentane, similarly of the reactants, by clogging the entrances to the micropores, as demonstrated in the case of the deSi-ZSM-5 sample.

**Figure 2:**
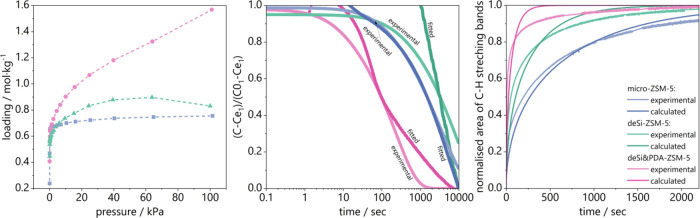
Neopentane sorption results over micro-ZSM-5, deSi-ZSM-5, and deSi&PDA-ZSM-5; (left) isotherms of neopentane sorption derived from volumetric experiments, (middle) adsorption kinetics from the first point of isotherm with data fitted automatically with BEL-Master software according to the Crank, and (right) normalized area of C–H stretching bands derived from RS FT-IR spectroscopy results with data fitted based on the Crank equation theory.

The mesoporosity of the catalysts and diffusion time constants were further assessed in kinetic experiments of customized neopentane sorption (Figure 2). The accumulation of neopentane on the surface of catalysts was followed in two modes: the volumetric one was dedicated to obtaining the isotherm of neopentane sorption, and the kinetic one to follow neopentane accumulation on the catalyst’s surface in the function of time. The former informed on the amount of external surface and meso/macroporosity accessible to neopentane while the latter on the diffusion of neopentane inside zeolite channels. The isothermal adsorption of neopentane proves the distinctly high sorption capacity of branched neopentane for deSi&PDA-ZSM-5. For the NaOH-derived sample, the increase in sorption capacity compared to micro-ZSM-5 is moderate. Further, both modified samples present inferior adsorption of neopentane at a lower pressure range, corresponding with lowered micropore volume after desilication. This is not surprising, as top-down methods of hierarchization are based on the development of mesoporosity at the expense of microporosity. Nevertheless, at least for neopentane sorption, a less evident drop in the low-pressure range is found in the desi&PDA-ZSM-5 hierarchical sample. The predominance of NaOH&TBAOH-derived samples is seen when diffusion rate constants calculated from the adsorption kinetics of neopentane are assessed (Figure 2 middle, Table 3). The diffusion time constant (D/r^2^, s^–1^; Table 3) for the deSi&PDA-ZSM-5 sample is an order of magnitude higher than for other samples.

**Table 3:** Diffusion Time Contents of Neopentane Derived from Kinetic Adsorption Measurements and Spectroscopic Studies (60 °C, 520 Pa) as well as from GCMC Simulations of Parent and Modified Samples

	diffusion time constant [s^–1^] derived from kinetic adsorption measurements	diffusion time constant [s^–1^] derived from FT-IR spectroscopy studies		
	520 Pa	520 Pa	diffusion time constant [s^–1^] derived from MD simulations	self-diffusion coefficient [cm^2^·s^–1^] derived from MD simulations
micro-ZSM-5	1.57 × 10^–5^	1.68 × 10^–4^	2.36 × 10^–4^	9.43 × 10^–14^
deSi-ZSM-5	0.93 × 10^–5^	3.31 × 10^–4^	5.13 × 10^–3^	2.05 × 10^–12^
deSi-ZSM-5&Ac		9.48 × 10^–4^		
deSi&PDA-ZSM-5	29.4 × 10^–5^	12.3 × 10^–4^	29.8 × 10^–3^	11.9 × 10^–12^
deSi&PDA-ZSM-5&Ac		21.3 × 10^–4^		

The diffusion of neopentane in studied zeolites was also assessed using rapid scan (RS) FT-IR spectroscopy studies. The RS FT-IR experiments coupled with a Crank solution for neopentane sorption allowed for determining the diffusion time constants^25^ (Figure 2 left side). The integral intensities of FT-IR spectra in the region of C-H stretching bands versus time were used as input data for the Crank solution, and the diffusion time constants were calculated. Applying the RS FT-IR technique allowed us to follow the diffusion phenomena in the appropriate time domain under conditions required for the respective process. The recently established FT-IR spectroscopy results analysis method delivers fast and valuable information on the neopentane diffusion ability in ZSM-5 zeolites. It can be easily transferred to other zeolitic materials and various probe molecules.^25^ The FT-IR spectroscopy and sorption results of neopentane are in high agreement and confirm the superiority of NaOH&TBAOH derived samples over those obtained solely from NaOH treatment. The treatment with acid additionally slightly improves the neopentane diffusion inside zeolite channels.

**Figure 3:**
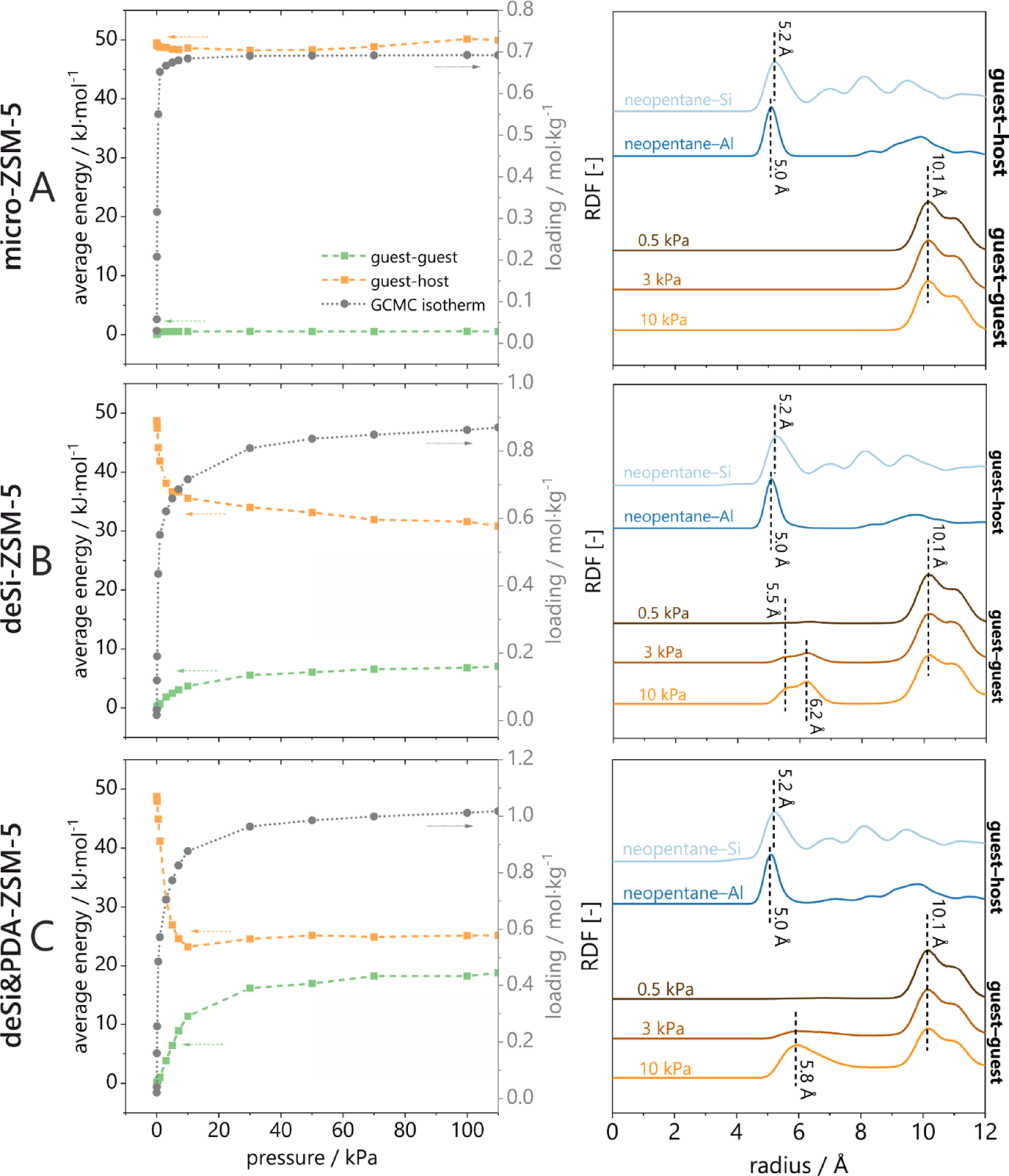
Guest–guest and guest–host adsorption energy contributions for the adsorption of neopentane in zeolite structures together with experimental and calculated isotherms (left side). Radial distribution functions showing distances from the neopentane center of mass to Si and Al atoms of ZSM-5 structures (right side); (A) micro-ZSM-5, (B) deSi-ZSM-5, (C) deSi&PDA-ZSM-5.

Additional insight into the mechanism of neopentane adsorption in zeolite pores is derived from Monte Carlo calculations performed in the grand canonical ensemble (GCMC). As one can observe in Figure 3, it is possible to reproduce the intensity and shape of the steep isotherm obtained in volumetric experiments of neopentane sorption. In some cases, the high-pressure range was not adjusted in the calculations as the adsorption on the external surface of grains is impossible to track in the GCMC calculations. The GCMC simulations mimic the behavior of the molecules only inside the pores, either micro- or mesopores. The analysis of the adsorption energy contributions shows that in the case of the unmodified micro-ZSM-5 zeolite, all the adsorption energy comes from interactions with the zeolite structure, reaching a value of about 50 kJ/mol. For the other two structures, interactions between the neopentane molecules play a large part, especially for the deSi&PDA-ZSM-5 sample with ink-bottle pores (Figure 3C), due to the larger mesopore space and the fact that a higher number of molecules can fit inside, unlike in the microporous counterpart. However, interactions with the host still predominate for all of the studied samples. This behavior is confirmed by the analysis of radial distribution functions (RDF, Figure 3 right side), where it is presented that the neopentane molecules are separated by a distance of 10 Å for the micro-ZSM-5 structure and both the deSi-ZSM-5 (Figure 3B) and deSi&PDA-ZSM-5 (Figure 3C) samples approach a distance of about 5–6 Å. For all models, the distances from Si and Al atoms are the same: 5.2 and 5.0 Å, respectively, which proves slightly better interactions with aluminum atoms. Despite the differences at higher pressures, the order of filling adsorption sites for all structures is also the same: at low pressures, neopentane molecules first occupy adsorption sites in the vicinity of aluminum atoms. Only at increased pressure do they fill the places in the surface pores and ink-bottle pores. Considering that the NaOH&TBOH derived samples provide both types of porosity, the one on the external surface and the one constrained, it can be stated that both mechanisms of pores filings will be found for this sample.

**Figure 4:**
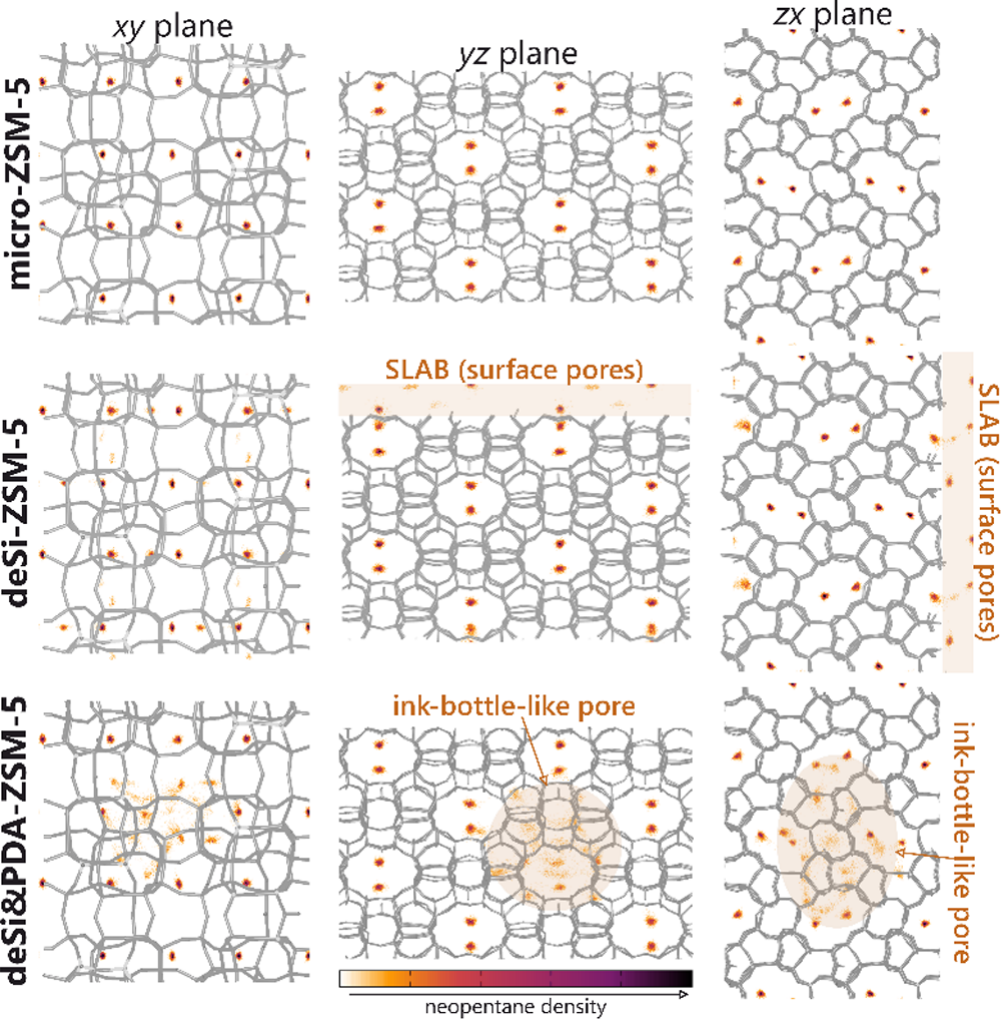
Average occupation profiles of neopentane adsorption in the ZSM-5 samples. The schemes of the structure and color scale (representing occupancy increasing from left to right) are also included.

The analysis of the average occupation profiles (Figure 4), showing the location of the centers of mass of neopentane in the pores of the materials in three projections, confirms all previously obtained information. It can be seen that guest molecules prefer places where aluminum atoms are located. Surface pores (deSi-ZSM-5) are located on both sides, on the slab surface and on hydrogen-terminated sites, which automatically reduces the distance between the molecules; this was observed with energy contributions and RDFs. In the case of ink-bottle pores (deSi&PDA-ZSM-5), more neopentane molecules enter the pore (where the entrance diameter was about 7 Å). They are located inside, thus increasing the energy of interaction with each other.

Diffusion time constants for all structures are calculated based on molecular dynamics simulations. Comparing the values obtained experimentally with those calculated by the MD method (Table 3), it can be seen that the order of magnitude is the same for the microporous zeolite micro-ZSM-5, which confirms the model’s compliance with the experiment. For the deSi-ZSM-5 and deSi&PDA-ZSM-5 models, the values are only an order of magnitude lower, which, however, can be considered entirely consistent due to the inevitable discrepancies, mainly due to the imperfections of the computational models. Moreover, it should be emphasized that the experiments measure molecular uptake on a macroscopic scale, and therefore, macroscopic factors may influence it. Analyzing the theoretical values, it can be concluded that due to the introduction of the surface and the ink-bottle pores, neopentane particles can diffuse more freely in the microporous system, which is reflected in the values of their diffusion time constants. The effect of additional porosity development was most strongly reflected in values found for deSi&PDA-ZSM-5, again confirming the superiority of this type of mesoporosity in hydrocarbons diffusion.

### Catalytic Cracking of Plastics over Microporous and Hierarchical Zeolites

**Figure 5:**
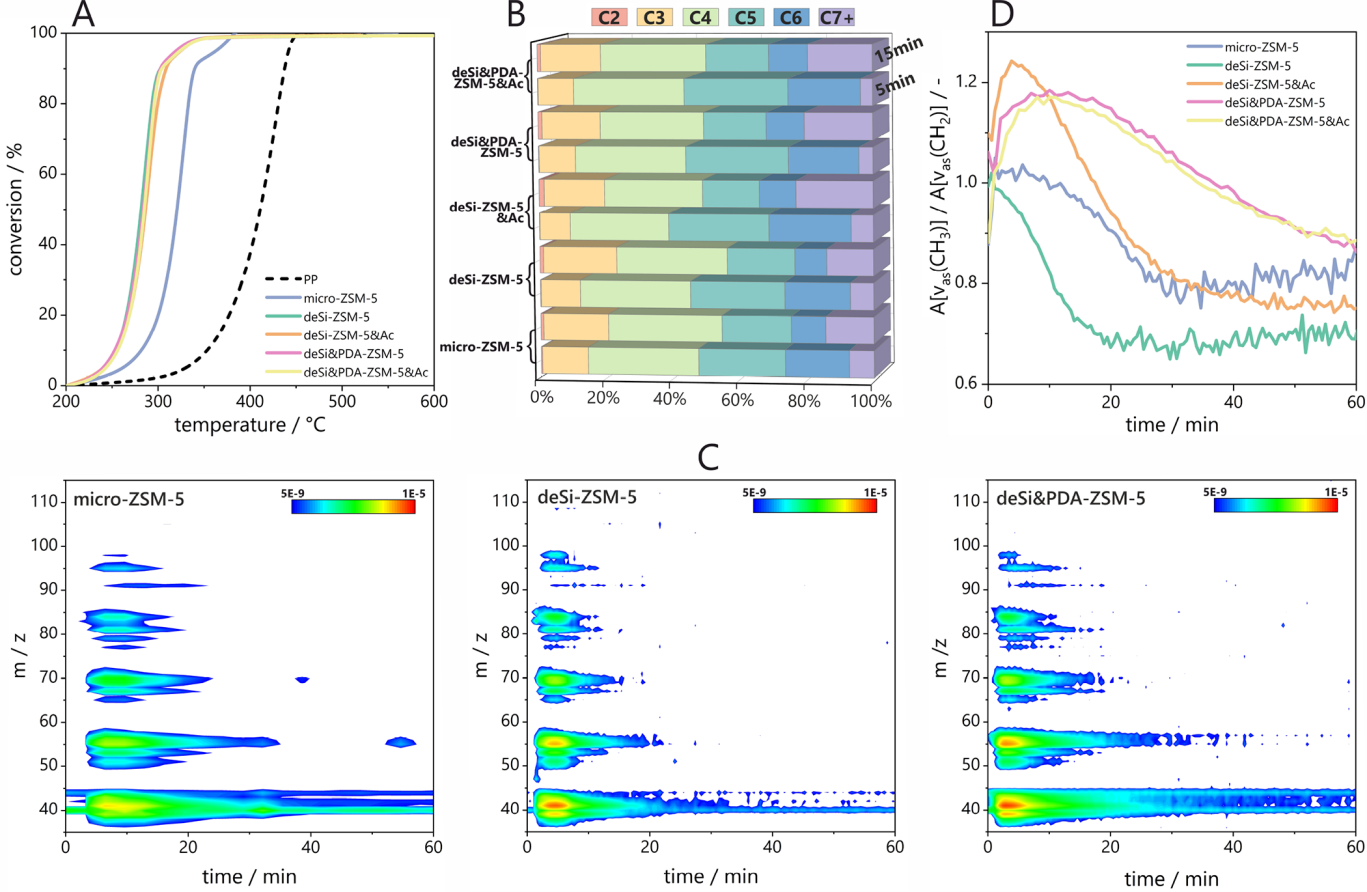
Catalytic results of PP cracking over studied catalysts. (A) Conversion curves derived from TG results, (B) selectivity of catalysts after 5 and 15 min of cracking process, (C) mass spectrometry results for selected samples in the form of 2D maps with time (min) and *m*/*z* ratio on axes; the color scale indicates the signal from mass spectrometer (arb. u.). (D) the CH_3_/CH_2_ band intensity ratio derived from *operando* FT-IR studies as a function of time during PP cracking.

The cracking activity is evaluated in two separate modes: one based on thermogravimetric experiments with increasing temperature under nitrogen flow and the spectroscopic one with *operando* FT-IR spectroscopy coupled with chromatographic and mass spectrometric analysis under constant temperature and the nitrogen flow. The catalyst and polypropylene (PP) were mixed, and a self-supported pellet was used in both studies. The thermogravimetric analyses (Figure 5A) indicate that all hierarchical zeolites performed higher than microporous zeolites in the cracking of PP. The microporous zeolite shifts the temperature of 50% conversion from 387 to 320 °C, while hierarchical zeolites lower it further to the 281–287 °C range. Additional information can be withdrawn from the curve shape at a high conversion range, where the curve slope is decreased. At the final stages of PP cracking, the catalysts are partially deactivated, and coke formation occurs, taking from the curve slope slightly higher coke formation accompanied by hierarchical zeolites. A more detailed analysis of coke will be provided in further sections.

GC-MS analysis of effluent gases and direct analysis of FT-IR spectra obtained from *operando* experiments provide additional insight into microporous and hierarchical zeolite cracking activity. The cracking over the micro-ZSM-5 zeolite results in the stable production of C_4_ hydrocarbons, while the C_3_ and C_7+_ hydrocarbons increase with time at the expense of C_5_ and C_6_ compounds (Figure 5B). The observed trends find reflection in all studied samples. However, the deSi&PDA-ZSM-5 samples offer the lowest amount of C_7+_ hydrocarbons at the initial stage of the cracking reaction. Nevertheless, the parent sample’s pure microporous character offers the propylene’s highest selectivity. Thus, the microporous character is desirable for the formation of propylene, while C_5_–C_6_ hydrocarbons result from a shorter diffusion path and higher accessibility to micropore entrances. Most of the C_3_–C_4_ formed hydrocarbons (>85%) for all studied zeolites are olefins, an expected consequence of polypropylene cracking. The discussion of the selectivity of PP cracking requires the combination of the porosity and acidity features, both significantly altered by the desilication process. For this reason, the share of the C_7+_ fraction could serve as an indicator of the effectiveness of the cracking processes. The zeolites characterized by high strength and at the same time limited accessibility of the sites located in micropores (micro-ZSM-5 and deSi-ZSM-5) present similar catalytic performance. The diminished accessibility in deSi-ZSM-5 results from an Al-rich shell, which importantly restrains the diffusion of reactants, by clogging the entrances to the micropores (Tables 2 and 3). The cleaning of zeolite micropores from Al-residue shorten time of residence of reactants in microspores and as a result the deSi-ZSM-5&Ac provides high selectivity to C_7+_ products. Open mesoporosity in NaOH-treated materials provides a different environment for reactants than constrained mesoporosity in NaOH&TBAOH-leached materials. Even though the latter possesses the acid sites of higher accessibility and lower strength than deSi-ZSM-5&Ac they still offer the lower C_7+_. The reason for that is the longer residence time facilitating recracking processes due to constrained mesoporosity.^33^ Further, it is generally anticipated that constrained porosity is responsible for advanced coking.^34^ In the case of NaOH&TBAOH-modified samples, the proper adjustment of acid strength and accessibility of acid sites also favors the secondary cracking reaction and hampers the coking process.

The coking process observed during thermogravimetric analysis is also reflected in the selectivity change with reaction time. The longer the reaction proceeds, the higher the share of heavier hydrocarbons found; this is related to the lower activity of the catalysts resulting from partial coking. The mass spectroscopy provides a continuous analysis of major reaction products (Figure 5C) that can be identified as follows: C_3=_ (*m*/*z* = 39, 41), C_4_ (*m*/*z* = 43), C_4=_ (*m*/*z* = 39, 41), C_5_ (*m*/*z* = 43), C_5=_ (*m*/*z* = 42, 55), C_6_ (*m*/*z* = 43, 57), C_6=_ (41, 56), C_7_ (*m*/*z* = 43, 57, 71), C_7=_ (*m*/*z* = 41, 55, 56, 69, 83), C_8_ (*m*/*z* = 43, 57, 71), C_8=_ (*m*/*z* = 41, 43, 55, 56, 69,70, 83), benzene (*m*/*z* = 78), toluene, ethylbenzene, xylenes, propylbenzene (*m*/*z* = 91), and mesitylene (*m*/*z* = 105). First, the majority of cracking occurs during the first 20 min of the process at isothermal conditions. However, for micro-ZSM-5 and deSi&PDA-ZSM-5 samples, the cracking was elongated in time with a higher share of products of lower carbon number. Based on the mass spectrometry results, olefins are the major formed products, as *m*/*z* = 39, 41, 55, 56, 69, and 83, which are the highest intensity during the reaction. It can also be noticed that the desilication with NaOH led to increased intensity of *m*/*z* above 100. Thus, heavier hydrocarbons are expected to be formed on this material. The formation of aromatics identified by *m*/*z* = 91 occurs with a time shift; therefore, the first cracking of polypropylene chains is necessary to form aromatics from shorter hydrocarbons.

An additional insight can be withdrawn based on the results of FT-IR spectroscopy. The ratio of bands representative for CH_3_ and CH_2_ groups (CH_3_ = 2960 cm^–1^, CH_2_ = 2925 cm^–1^) of hydrocarbons adsorbed on catalysts surface provides information on the susceptibility of PP end-chain cracking mechanism over catalysts (Figure 5D). From the fwhm of curves representing the CH_3_/CH_2_ ratio, it is clear that the cracking process over catalysts with ink-bottle-like-shaped mesopores proceeds longer than that on the catalysts with surface mesopores; the same was found based on mass spectra analysis. Additionally, the higher values of CH_3_/CH_2_ confirm the presence of longer chain hydrocarbons over the surface of deSi&PDA-ZSM-5 and deSi&PDA-ZSM-5&Ac catalysts, which were also found in the results of chromatographic analysis. The acid treatment significantly affected the CH_3_/CH_2_ ratio curve for the NaOH-derived sample, while for NaOH&TBAOH, no changes were observed. Furthermore, the selectivity of deSi-ZSM-5&Ac resembles those found for deSi&PDA-ZSM-5 and deSi&PDA-ZSM-5&Ac catalysts. Thus, the catalysts desilicated in the presence of NaOH&TBAOH are expected to be free of Al-rich shell^6,11^ that needs to be removed from NaOH-derived zeolites to reach their full catalytic potential.

### 2D COS Analysis of Plastics Cracking over Microporous and Hierarchical Zeolites

**Figure 6:**
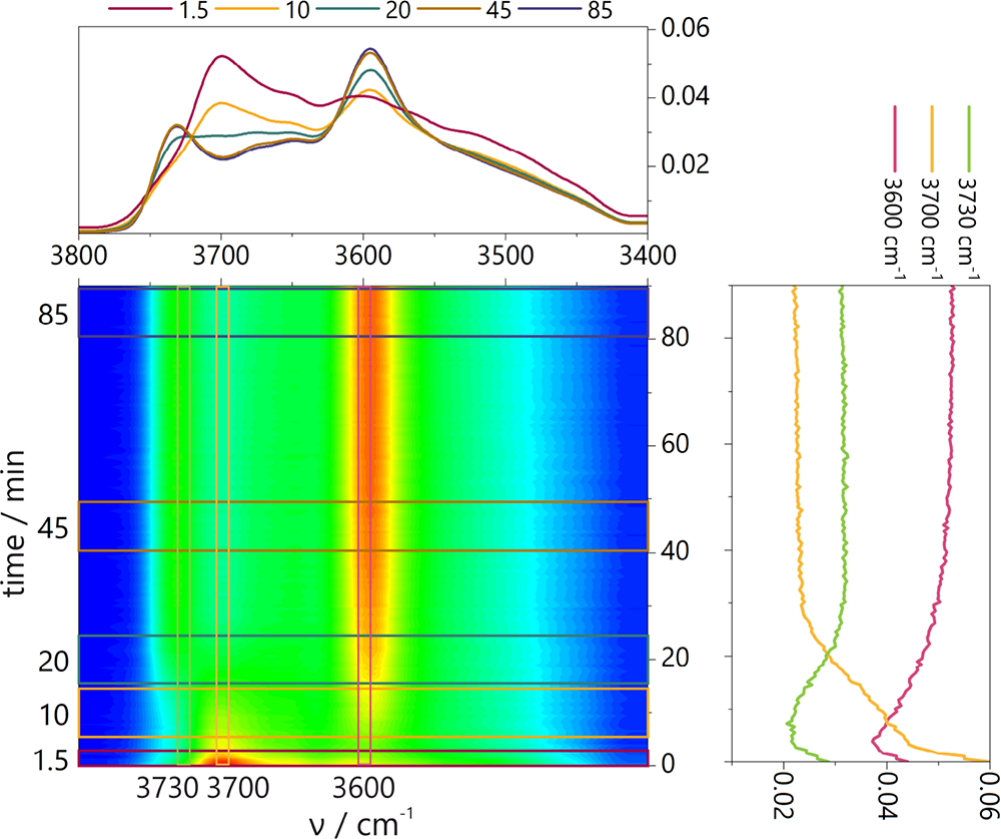
Top-down projection maps of FT-IR spectra with band traces of Si(OH) (3730 cm^–1^), Si(OH)Al (3600 cm^–1^), and O–H from H_2_O (3700 cm^–1^) during cracking of polypropylene over micro-ZSM-5 catalyst in the quartz *operando* cell.

Polypropylene (PP) cracking is followed by *operando* FT-IR spectroscopy under constant temperature and nitrogen flow. The catalyst and PP were mixed, and a self-supported pellet was placed in a spectroscopic cell. Upon cracking of plastic over zeolite samples, the restoration of silanol Si(OH) (3735 cm^–1^) and acidic Si(OH)Al groups (ca. 3600/3585 cm^–1^) is observed, while the band of residual water is diminished (3700 cm^–1^) (Figure 6).

The set of 2D CoS maps for all studied catalysts was prepared from FT-IR spectra collected during the cracking of polypropylene. The 2D CoS synchronous (sync) and asynchronous (async) maps were divided into ranges 3800–3400, 3050–2700, and 1650–1300 cm^–1^ for scrutiny of the O–H groups, C–H stretching bands, C–C stretching, and C–H deformation bands, respectively. For clarity, only selected maps will be shown, while all maps are placed in the Supporting Information (Figures S5–S9). The asynchronous correlations in the range 3800–3400 cm^–1^ inform on the preference of Si(OH)Al (ca. 3600 cm^–1^) groups over Si(OH) (ca. 3730 cm^–1^) ones during restoration; this effect is more pronounced for hierarchical zeolites than for micro-ZSM-5 as the level of correlation is distinctly higher for all modified samples (Figures S5–S9A,A′). This trend correlates with the increased accessibility of the Si(OH)Al groups and, thus, the facile release of cracking products from acidic centers without poisoning or side reactions. Even though the Si(OH) groups are restored after plastic cracking, this process is delayed compared with the restoration of the Si(OH)Al sites. The Si(OH) groups of low, if any, acidity can serve as centers for coke species occupancy. Thus, their restoration might be delayed. The 3650 and 3673 cm^–1^ bands of the Al(OH) groups from EFAl species are also found for hierarchical zeolites. However, those groups seem to be consumed during the catalytic process. This consumption of OH groups over EFAl species proceeds before the restoration of the Si(OH) groups. Thus, the OH groups of EFAl species are either occupied during the catalytic cracking of plastics or are centers of adsorption of products remaining on the catalyst surface, i.e., precursors of coke species. Indeed, the micro-ZSM-5 and NaOH-derived samples present a lower amount of coke residue after cracking of PP in thermogravimetric studies (Table 4). As mentioned, the Si(OH) groups formed on the surface of NaOH&TBAOH modified samples are prone to interaction with neopentane and thus most probably with other hydrocarbons and might serve as centers of sorption for coke precursor and species. In addition, the Al-rich shell can importantly restrain the diffusion of neopentane and thus the reactants by clogging the entrances to the micropores, as demonstrated in the case of the deSi-ZSM-5.

**Table 4:** TG-Derived Coke Content from Spent Catalysts after Catalytic Tests in Thermogravimetric Mode

	coke content %
micro-ZSM-5	0.65
deSi-ZSM-5	1.49
deSi-ZSM-5&Ac	1.19
deSi&PDA-ZSM-5	1.33
deSi&PDA-ZSM-5&Ac	1.33

**Figure 7:**
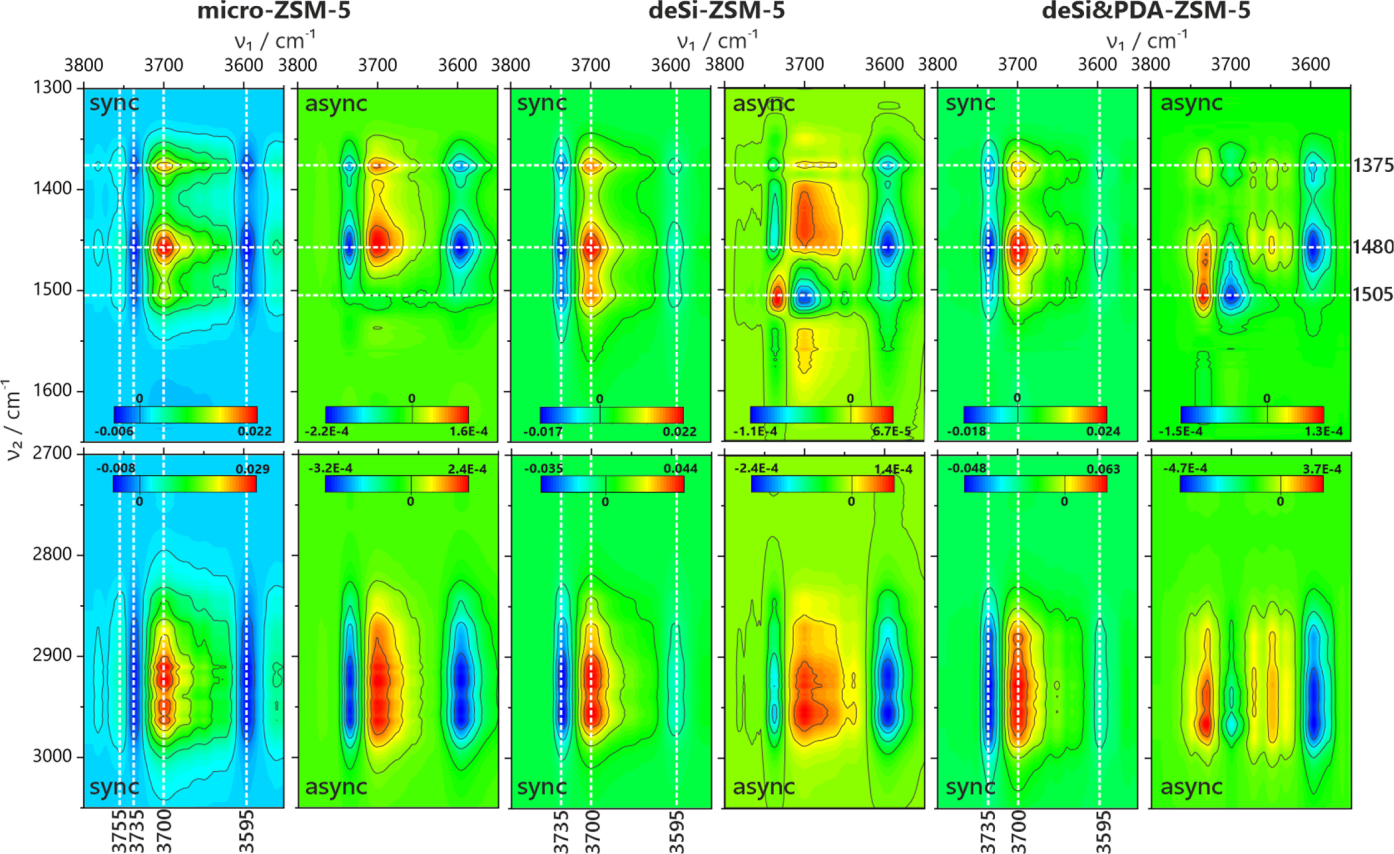
2D CoS maps for *operando* FT-IR spectroscopic studies of PP cracking over selected catalysts. (Left) micro-ZSM-5, (middle) deSi-ZSM-5, (right) deSi&PDA-ZSM-5. The maps present correlations between the 3800–3550 cm^–1^ region with two other regions: 3050–2700 and 1650–1300 cm^–1^. Synchronous and asynchronous maps are marked as sync and async, respectively.

**Figure 8:**
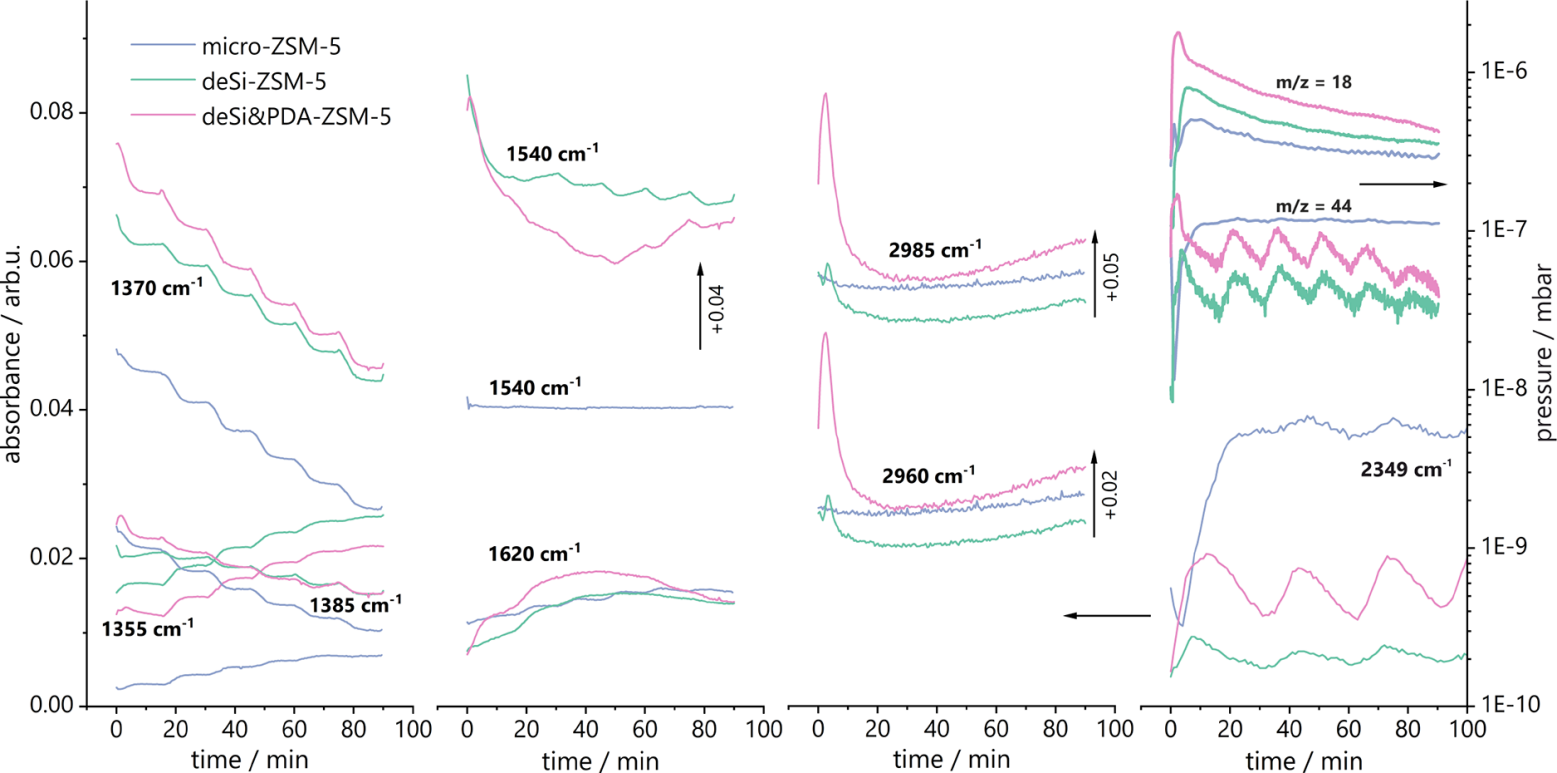
Traces of IR bands (1355, 1370, 1385, 1540, 1620, 2349 (CO_2_), 2960, 2985) and mass spectrum signal for *m*/*z* = 44 (CO_2_) and 18 (H_2_O) during coke burning-off after catalytic cracking of PP over studied samples.

Figure 7 presents that upon cracking, the restoration of Si(OH)Al (ca. 3610 cm^–1^) and Si(OH) (3734 cm^–1^) groups (3800 – 3400 cm^–1^) is observed, and it correlates with the consumption of the C–H (3050–2700 cm^–1^) and C–C (1650–1300 cm^–1^) bands representative for polypropylene decomposition as well as with the 3700 cm^–1^ band of residual amounts of desorbing water Figures S5–S9B,B′,D,D′). The correlations are the positive or negative peaks at the 2D maps of regions representative of the O–H, C–C, or C–H bond vibrations. On the synchronous maps, the positive correlation indicates the same direction of changes in band intensity, either an increase or decrease of both bands. When the correlation peak between two bands on a synchronous map is negative, their intensities change in opposite directions. Further, if the asynchronous correlations are included in the analysis, then the order of changes can be found. If the peaks on both types of maps are of the same sign, the peak at the higher wavenumber (ν_1_) precedes the one at the lower wavenumber (ν_2_). If the peaks have different signs, one positive and the other negative, then the peak at the higher wavenumber (ν_1_) follows the one at the lower wavenumber (ν_2_). At the cracking initiation stage, the restoration of Si(OH)Al groups precedes the consumption of C–H and C–C bands representative of polypropylene (Figure 8). This is seen as a negative correlation with the 3610 cm^–1^ band on synchronous and asynchronous maps; the Si(OH)Al group band is of higher wavenumber than the C–H and C–C bands; thus, it changes as the first. Afterward, when the cracking process is continued, the order of events is reversed, and the consumption of polypropylene bands precedes the Si(OH)Al groups restoration (Figure S10) as the sign of correlations for the 3610 cm^–1^ band is negative on the synchronous map but positive on the asynchronous map. This should be related to realizing the acidic Si(OH)Al groups from water interaction and their engagement in polypropylene cracking. The later stages of polypropylene cracking also show a higher correlation between the O–H groups and bands around 1520–1480 cm^–1^. The drop in intensity of 1520–1480 cm^–1^ bands precedes the increase in intensity of the Si(OH)Al groups at 3600 cm^–1^; this might be related to the formation of heavier hydrocarbon compounds in later stages of reaction as 1520–1480 cm^–1^ bands might be indicative for aromatics^35^ and their removal from zeolite surface during cracking preceding the realization of the Si(OH)Al groups. Indeed, the mass spectrometry results showed that the *m*/*z* = 91 signal was found later in reaction time compared to other products of PP cracking. The restoration of Si(OH) groups occurs during the whole process after the degradation of polypropylene. However, for micro-ZSM-5 and deSi-ZSM-5 samples, Si(OH) groups are less prone to interact with polymer chains as the Si(OH) restoration occurs before consuming the C–H and C–C bands representative for polypropylene. Thus, desilication using PDA and/or acid washing leads to external surfaces covered with Si(OH) groups prone to interaction with polymer hydrocarbon chains. This strongly relates to the behavior found for neopentane interaction with Si(OH) groups. Only the silanols of NaOH&TBAOH-derived samples were prone to interaction with neopentane. Thus, neopentane and the described methodology can assess the diffusivity and predict catalytic activity in hydrocarbon cracking reactions. The difference between the order of changes of Si(OH)Al and Si(OH) groups between the samples can be related to their location. The polypropylene must be first partially cracked to smaller hydrocarbon chains able to interact with Si(OH)Al groups located inside micropores, even those Si(OH)Al that are highly accessible. Furthermore, only the Si(OH) groups generated during the desilication using PDA and/or acid wash are involved in cracking reactions; those found in micro-ZSM-5 or generated during the desilication with NaOH are not. Finally, the role of Si(OH) groups is important in the initial reaction stage, while later on, the Si(OH)Al groups are the dominant ones.

The 2D CoS analysis of IR spectra also allowed us to follow the order of changes of bands representative for polypropylene (Figures S5–S9C,C′,E,E′,F,F′). For the C–H stretching (3050–2700 cm^–1^) and C–C/C–H deformation band (1650–1300 cm^–1^) regions, the 2D CoS maps showed strong positive correlations resulting from their successive decreasing intensity during the cracking of polypropylene. The only negative correlation was found for the band at 1620 cm^–1^, suggesting the formation of coke precursors of an aromatic/polyolefinic nature. The decrease in the broad band at 1520–1480 cm^–1^ precedes the drop in intensity of other C–C/C–H deformation bands. Thus, the representative species are the least stable ones inside zeolite channels, as this region might suggest the presence of *p*-xylene (1515 cm^–1^), toluene (1495 cm^–1^), and benzene (1478 cm^–1^).^35^ Furthermore, the changes in the range of C–H stretching bands (3050–2700 cm^–1^) are subsequent to the decrease of the broad band at 1520–1480 cm^–1^ and the bands at 1400–1350 cm^–1^. This observation can be directly related to the progressive cracking reaction of C–C bonds in polypropylene and the removal of the formed products. In the overall reaction picture, C–C bond cracking precedes the changes in C–H bonds. This can also suggest that the isomerization or dehydrogenation process is secondary to the cracking.

### Coke Analysis: Spectroscopic and Thermogravimetric Analyses

The final assessment of the suitability of desilication with quaternary ammonium cations as pore-directing agents was performed bearing the coke nature and content after cracking polypropylene. The coke properties were studied during thermogravimetric analysis and thermoprogrammed oxidation in FT-IR&MS spectroscopic studies after the cracking of polypropylene.

The thermogravimetric analysis shows that the micro-ZSM-5 zeolite requiring the highest temperatures for PP cracking offered the highest coke resistance (Table 4). The development of secondary mesoporosity led to higher coke formation; thus, the silanol groups populating the external surface might be responsible for retaining the coke precursors on the external surface.^36^ Before the cracking of polypropylene inside micropores, the melting is required,^37^ and initial cracking is required on external sites and/or in pore mouths. Hierarchical zeolites in thermogravimetric assessment of catalytic activity required lower temperatures for PP cracking due to high external surface area and thus better contact between the catalyst and feed. The lower temperatures of initial cracking on the external surface of catalysts may lead to heavier hydrocarbons retaining on the outer parts of grains. Thus, the coke formation on the external surface may be enhanced for hierarchical materials with secondary mesoporosity.^4^

The analysis of FT-IR spectra during thermoprogrammed oxidation from 300 to 550 °C showed that the intensities of IR bands at 2960, 2925, 1620, 1540, 1385, 1375, and 1355 cm^–1^ change gradually with temperature and time (Figure 8). The 1385 and 1375 cm^–1^ bands representative for the CH_3_ and CH_2_ deformation vibrations decrease strongly during oxidation, accompanied by an increase of 1355 cm^–1^ band possibly related to the O–H bending vibrations of hydroxyl groups of partially oxidized coke species. The 2960 and 2930 cm^–1^ bands representative for CH_3_ and CH_2_ stretching vibrations strongly decreased during the first 20 min of the oxidation process (up to 350 °C), most probably related to removing soft coke reaching in hydrogen. The same trend is found for the 1540 cm^–1^ band representative of conjugated olefinic species.^38^ The 1620 cm^–1^ band is found to increase slightly until the temperature of 450 °C; thus, it is anticipated that the methylsubstituted aromatics representative of this band are formed during the temperature increase from residual olefinic species. At temperatures above 500 °C, a decrease of 1620 cm^–1^ is found, while the bands of 2960, 2930, and 1540 cm^–1^ slightly increase. Thus, the cyclization and aromatization processes occurring in lower temperatures are followed by the cracking of cyclic/aromatic species at higher temperatures and their removal from the catalyst’s surface. The thermoprogrammed oxidation results in the formation of CO_2_ molecules representative of the band at 2349 cm^–1^; the highest intensity is found for the micro-ZSM-5 zeolite. The same observation for the micro-ZSM-5 catalyst is found by MS results (Figure 8, *m*/*z* = 44). Further, for the micro-ZSM-5, the lowest amount of water formed upon coke oxidation was observed (*m*/*z* = 18). Thus, the coke formed and oxidized in temperatures below 600 °C for micro-ZSM-5 is highly condensed and hydrogen deficient. For hierarchical zeolites, most importantly for deSi&PDA-ZSM-5 catalysts, the lower amount of CO_2_ and higher amount of H_2_O is released during coke burning-off. Thus, it is anticipated that the formed coke is more susceptible to oxidation even if it has a higher content, as found in the TG analysis for hierarchical samples.

**Figure 9:**
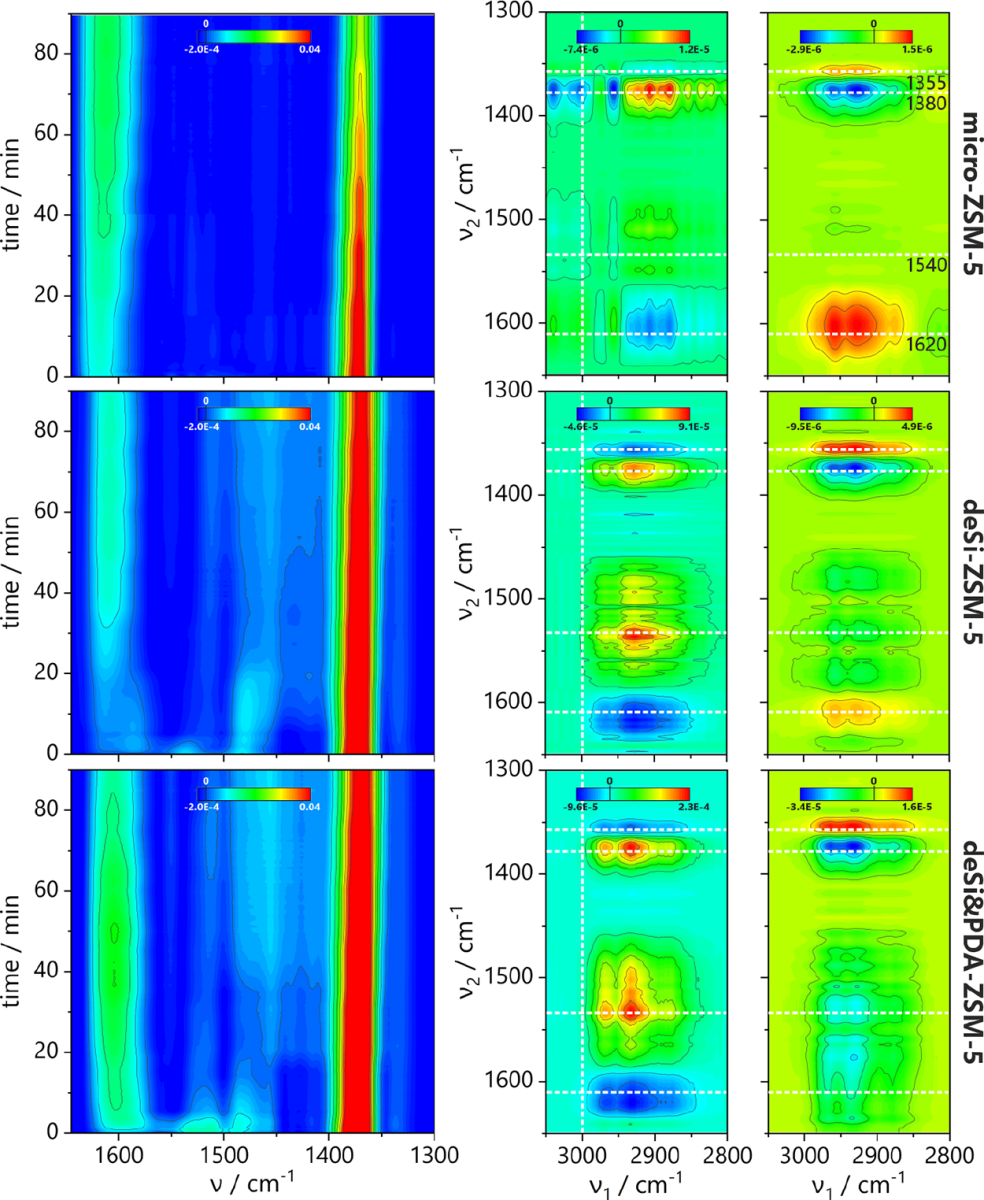
Top-down projection maps of FT-IR spectra in the 1650–1300 cm^–1^ region (left); 2D sync and async maps of spectral region characteristic for C–H/C–C stretching and deformation vibrations (right) resulting from the spectra collected during coke burning-off for selected studied catalysts.

Additional information on the nature of coke is provided by 2D CoS analysis of FT-IR spectra (Figure 9). The micro-ZSM-5 zeolite presented distinct behavior from all hierarchical samples. The correlation between the C–H bending bands at ca. 1380 cm^–1^ and the C–H stretching bands above 3000 cm^–1^ confirms the presence of methyl-substituted aromatic hydrocarbons as coke species. The correlation peaks in the 2D CoS maps show the coupling between the bands at 1620, 1540, 1385, 1375, and 1355 cm^–1^ and those below 3000 cm^–1^. The increase of band intensity at 1620 and 1355 cm^–1^ is accompanied by the decrease of bands located below 3000 cm^–1^ as the negative correlation peaks are found on 2D sync-maps for all samples. Similarly, the decrease of band intensity at 1385 and 1375 cm^–1^ is coupled with the latter, as the positive peaks are found on sync-maps at these positions. Additionally, the 2D async-maps present the peaks at identical locations but with opposite signs. The set of 2D CoS maps for the region 1650 × 1300 cm^–1^ also provides the opposite sign of peaks on 2D sync and async maps (Figure S11). Thus, according to Noda’s rules,^19^ the bands at a lower wavenumber change their intensity before those at a higher wavenumber on the FT-IR spectra. It is anticipated that the decomposition of −CH_3_ groups (1385–75 cm^–1^ bands) precedes the decomposition of conjugated olefinic species (1540 cm^–1^ band) and the formation of methyl-substituted aromatics 1620 cm^–1^ band). The last changes in the FT-IR spectra are seen in the region of C–H stretching bands of alkanes (bands below 3000 cm^–1^). The highest intensity of correlation peaks is found for deSi&PDA-ZSM-5, which suggests the highest rate of coke precursor formation. Nevertheless, based on FT-IR and MS analysis, the formed coke is of a rather olefinic nature, has a low C/H ratio, and is feasible for oxidation.

## Conclusions

The desilication using quaternary ammonium cations proved to be a suitable route for obtaining catalytically active materials for cracking polypropylene. The activity of hierarchical materials with constrained mesoporosity was slightly lowered in a manner of elongated time necessary for cracking of polypropylene; however, it resulted in a higher share of products with a lower carbon number. Further, the coke content was only slightly higher (1.33%) than that for materials with open mesopores after the required acid wash (1.19%). The hierarchical zeolites with constrained porosity proved to have distinctly higher sorption capacity for branched neopentane, which confirmed the superiority of the developed mesoporosity for hydrocarbon bonding. The surface of the mesopores formed with the assistance of a pore-directing agent was enriched in silanols that interacted with neopentane. Further, the location of strong Lewis acid sites from dehydroxylation was also affected by the presence of tetrabutylammonium cation, and only for NaOH-treated samples did it block the pore mouth entrances. The constrained mesopores offered higher diffusion rates for neopentane, which was confirmed in experimental and theoretical investigations. The two-dimensional correlation spectroscopy gave us additional insight from *in situ* and *operando* FT-IR spectroscopic investigations.

## Data Availability

Spectroscopic investigation of acidity, diffusion and activity of zeolites for polypropylene cracking (Original data) (Jagiellonian University Repository) https://uj.rodbuk.pl/dataset.xhtml?persistentId=doi:10.57903/UJ/N10OVI

## Supporting Information Available

Simulation details; X-ray diffraction patterns and nitrogen physisorption isotherms; FT-IR spectra of CO adsorbed at −130 °C on the zeolites studied; 2D CoS maps from RS FT-IR spectra registered during neopentane sorption overall the catalysts studied; 2D synchronous and asynchronous CoS maps from operando FT-IR spectroscopic studies of PP cracking over micro-ZSM-5, deSi-ZSM-5, deSi-ZSM-5&Ac, and deSi&PDA-ZSM-5; 2D CoS maps for sample deSi&PDA-ZSM-5 of spectra registered during cracking of PP after the period of initial cracking; and 2D CoS sync and async maps registered during coke burning off from studied samples (PDF)

## Notes

All the images that appear in Manuscript and Supporting Information files, including those in the TOC graphic, are original and created by the authors of this manuscript.
