# Supplementary material for: Constrained and Open Mesoporosity in Polypropylene Cracking: Insight From Spectroscopic Investigations of Acidity, Diffusion, and Activity
Source: Langmuir. 2024 Mar 23;40(13):6918–32. doi: 10.1021/acs.langmuir.3c03880 (PMC10993412; doi:10.1021/acs.langmuir.3c03880)
Supplement: Supplementary file 1 — la3c03880_si_001.pdf [file la3c03880_si_001.pdf]

Supporting Information for

Constrained and open mesoporosity in polypropylene cracking – insight from spectroscopic investigations of acidity, diffusion and activity

Karolina A. Tarach[1*], Gabriela Jajko[1,2], Miguel Palomino[3], Fernando Rey[3], Kinga Góra-Marek[1**]

[1] Faculty of Chemistry, Jagiellonian University in Kraków, Gronostajowa 2, 30-387 Kraków, Poland

[2] Doctoral School of Exact and Natural Sciences, Jagiellonian University in Krakow, Łojasiewicza 11, 30-348 Krakow, Poland

[3] Instituto de Tecnología Química, Universitat Politècnica de València – Consejo Superior de Investigaciones Científicas (UPV-CSIC), Avda. de los Naranjos s/n, 46022 Valencia, Spain

* karolina.tarach@uj.edu.pl

** kinga.gora-marek@uj.edu.pl

Number of pages: 13

Number of figures: 10

Number of tables: 2

A table of contents:

Experimental Section S2

Simulations details S2

Table S1. Neopentane and ZSM-5 forcefield parameters used for the Monte Carlo simulation S2

Table S2. Modified Lorentz-Berthelot mixing rules. S2

Figures S3

Fig. S1. X-ray diffraction patterns (left), nitrogen physisorption isotherms (right) and BJH pore size distribution (inset) for studied zeolites. S3

Fig. S2 FT-IR spectra of CO adsorbed at -130 ºC on zeolites after full saturation of Lewis acid sites. S4

Fig. S3 2D CoS maps (synchronous – left, asynchronous – right) from RS FT-IR spectra registered during neopentane sorption overall studied catalysts. Maps are presented in full scale. S5

Fig. S4 2D CoS maps of RS FT-IR spectra registered during neopentane sorption overall studied catalysts in the region of O-H stretching groups. S6

Fig. S5 2D CoS (synchronous – left, asynchronous – right) from operando FT-IR spectroscopic studies of PP cracking over micro-ZSM-5 zeolite. S7

Fig. S6 2D CoS (synchronous – left, asynchronous – right) from operando FT-IR spectroscopic studies of PP cracking over deSi-ZSM-5 zeolite. S8

Fig. S7 2D CoS (synchronous – left, asynchronous – right) from operando FT-IR spectroscopic studies of PP cracking over deSi-ZSM-5&Ac zeolite. S9

Fig. S8 2D CoS (synchronous – left, asynchronous – right) from operando FT-IR spectroscopic studies of PP cracking over deSi&PDA-ZSM-5 zeolite. S10

Fig. S9 2D CoS (synchronous – left, asynchronous – right) from operando FT-IR spectroscopic studies of PP cracking over deSi&PDA-ZSM-5&Ac zeolite. S11

Fig. S10 Set of 2D CoS maps for sample deSi&PDA-ZSM-5 of spectra registered during cracking of PP after the period of initial cracking, i.e. after total realising of Si(OH)Al groups from water adsorption. S12

Fig. S11 Set of 2D CoS sync- and async-maps in region 1650 – 1300 cm^{-1} registered during coke burning off from studied samples. S13

Experimental Section

Simulations details

Nonbonded interactions between guest molecules and the host framework were modelled using a Lennard-Jones potential:

$$U^{L-J}(r_{ij}) = 4\varepsilon_{ij}\left[\left(\frac{\sigma_{ij}}{r_{ij}}\right)^{12} - \left(\frac{\sigma_{ij}}{r_{ij}}\right)^{6}\right] + \frac{q_i q_j}{4\pi\varepsilon_0 r_{ij}} \qquad \text{(S1)}$$

where r_{ij} is distance between *i* and *j* atoms. For each MFI framework atom and adsorbates (pseudo)atoms ε and σ values from Table 1 were used, which were mixed using Lorentz-Berthelot rules:

$$\varepsilon_{ij} = \sqrt{\varepsilon_i \cdot \varepsilon_j} \qquad \text{(S2)}$$

$$\sigma_{ij} = \frac{\sigma_i + \sigma_j}{2} \qquad \text{(S3)}$$

Intramolecular bonded interactions within the molecules were also used:

1) Harmonic bonding potential:

$$U^{bond}(r_{ij}) = \frac{1}{2}\left(r_{ij} - r_{ij}^{0}\right)^2 \qquad \text{(S4)}$$

2) Harmonic bending potential:

$$U^{bend}(\theta_{ijk}) = \frac{1}{2}\left(\theta_{ijk} - \theta_{ijk}^{0}\right)^2 \qquad \text{(S5)}$$

3) Torsions TraPPE cosine series potential:

$$U^{torsion}(\phi_{ijkl}) = p_0 + p_1[1 + \cos\phi_{ijkl}] + p_2[1 - \cos 2\phi_{ijkl}] + p_3[1 + \cos 3\phi_{ijkl}] \qquad \text{(S6)}$$

where r_{ij}/r_{ij}^{0} is actual/equilibrium bond length, $\theta_{ijk}/\theta_{ijk}^{0}$ is actual/equilibrium bend angle, and ϕ_{ijkl} is actual dihedral angle.

Table S1. Neopentane and ZSM-5 forcefield parameters used for the Monte Carlo simulation

2,2-dimethylpropane (UA) – flexible model (GenericZeolites)			
Nonbonded interactions			
(pseudo)atom	ε/k_B [K]	σ [Å]	q [e^-]
CH_3_sp3	108.0	3.76	0.0
C_sp3	0.80	6.38	0.0
Harmonic bond		Harmonic bend	
r_{C-C} [Å]		θ_{C-C-C}[deg]	
1.54		109.47	
ZSM-5			
Nonbonded interactions			
(pseudo)atom	ε/k_B [K]	σ [Å]	q [e^-]
Si	22.0	2.30	-
O	53.0	3.30	-
Al	22.0	2.30	-
H	7.65	2.85	

Table S2. Modified Lorentz-Berthelot mixing rules.

neopentane (pseudo)atoms	ZSM-5 atoms	ε/k_B [K]	σ[Å]
CH_3_sp3	O	93.0	3.48
C_sp3		10.0	4.56

Figures

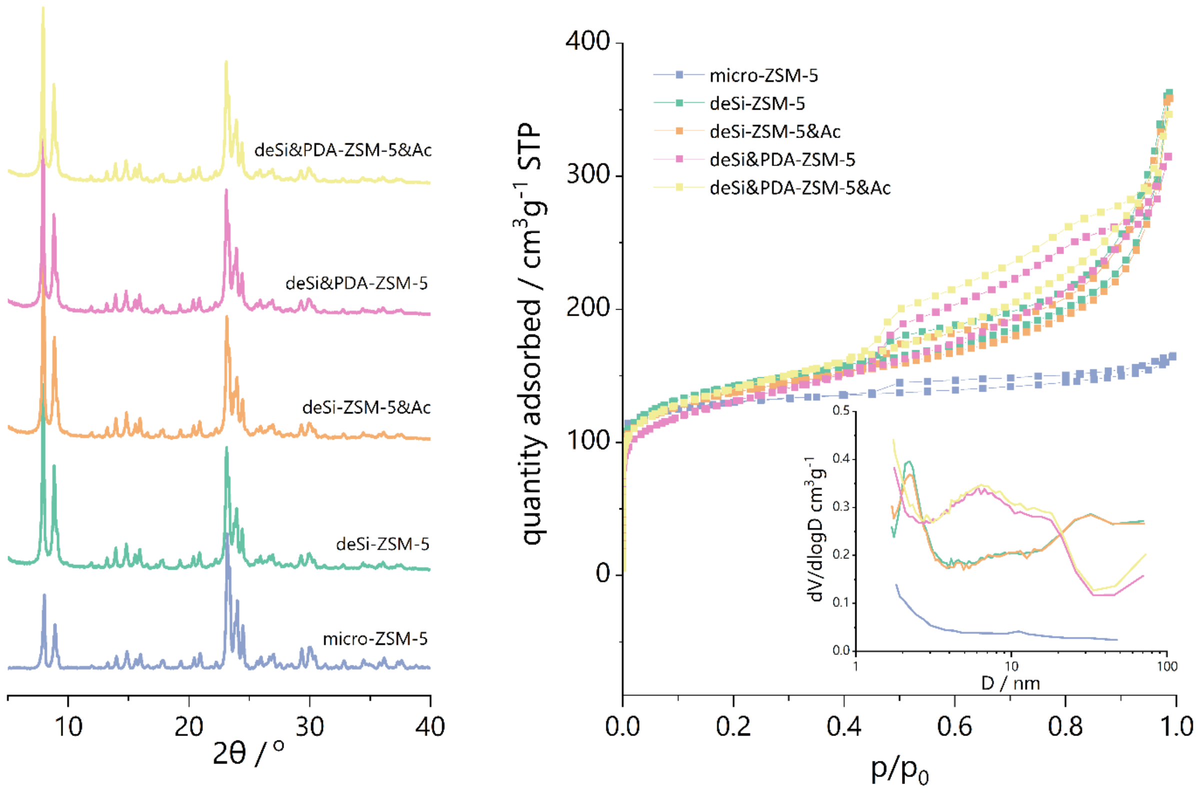

Fig. S1. X-ray diffraction patterns (left), nitrogen physisorption isotherms (right) and BJH pore size distribution (inset) for studied zeolites.

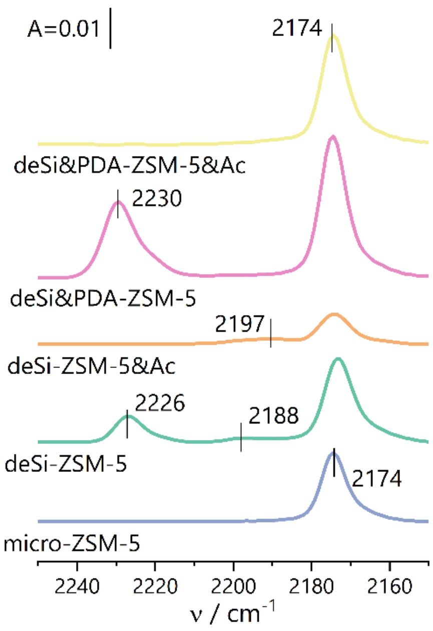

Fig. S2 FT-IR spectra of CO adsorbed at -130 ºC on zeolites after full saturation of Lewis acid sites.

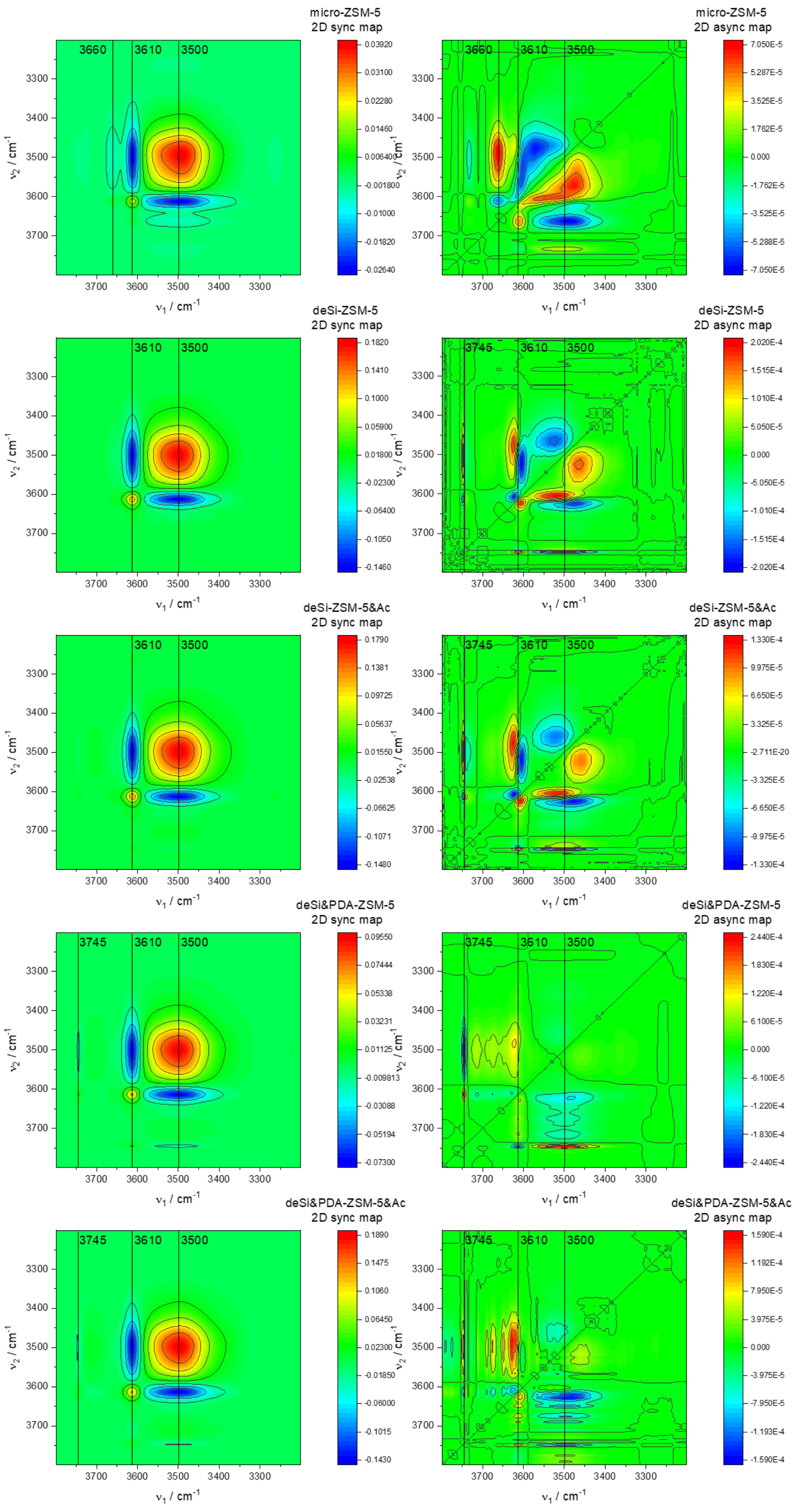

Fig. S3 2D CoS maps (synchronous – left, asynchronous – right) from RS FT-IR spectra registered during neopentane sorption overall studied catalysts. Maps are presented in full scale.

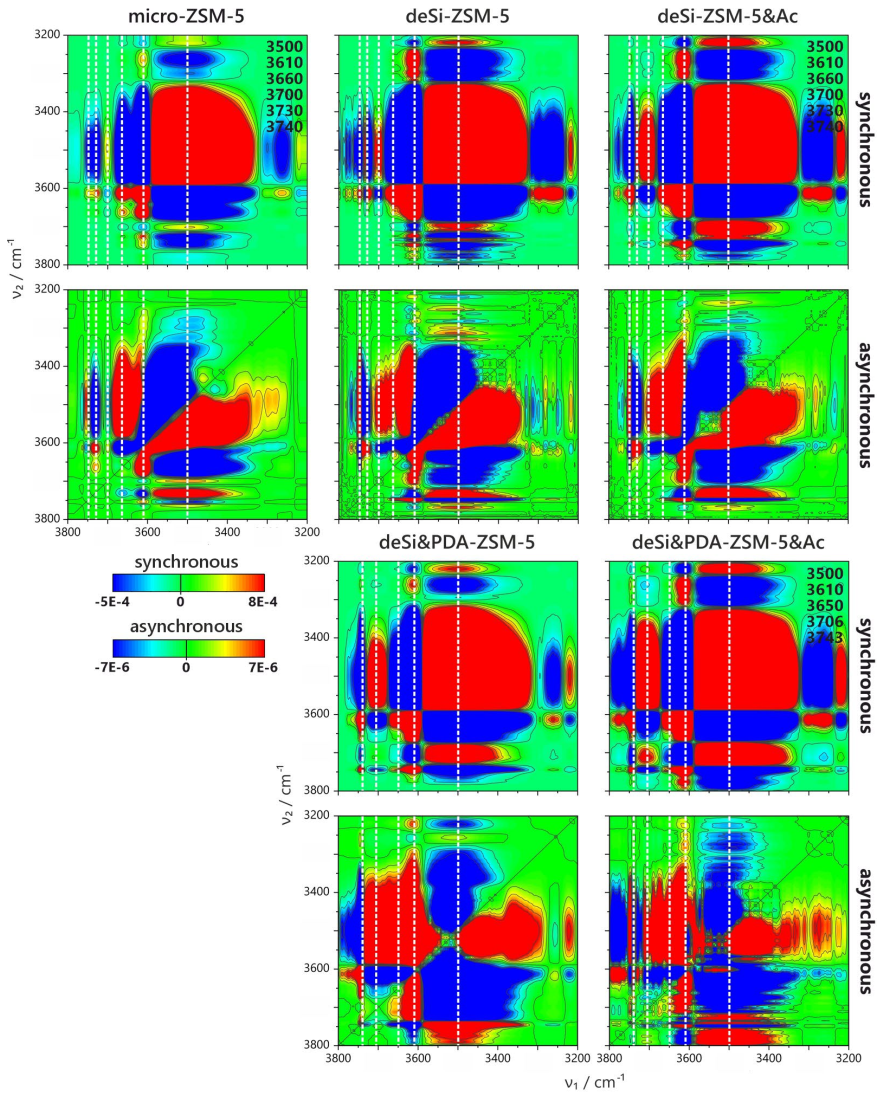

Fig. S4 2D CoS maps of RS FT-IR spectra registered during neopentane sorption overall studied catalysts in the region of O-H stretching groups.

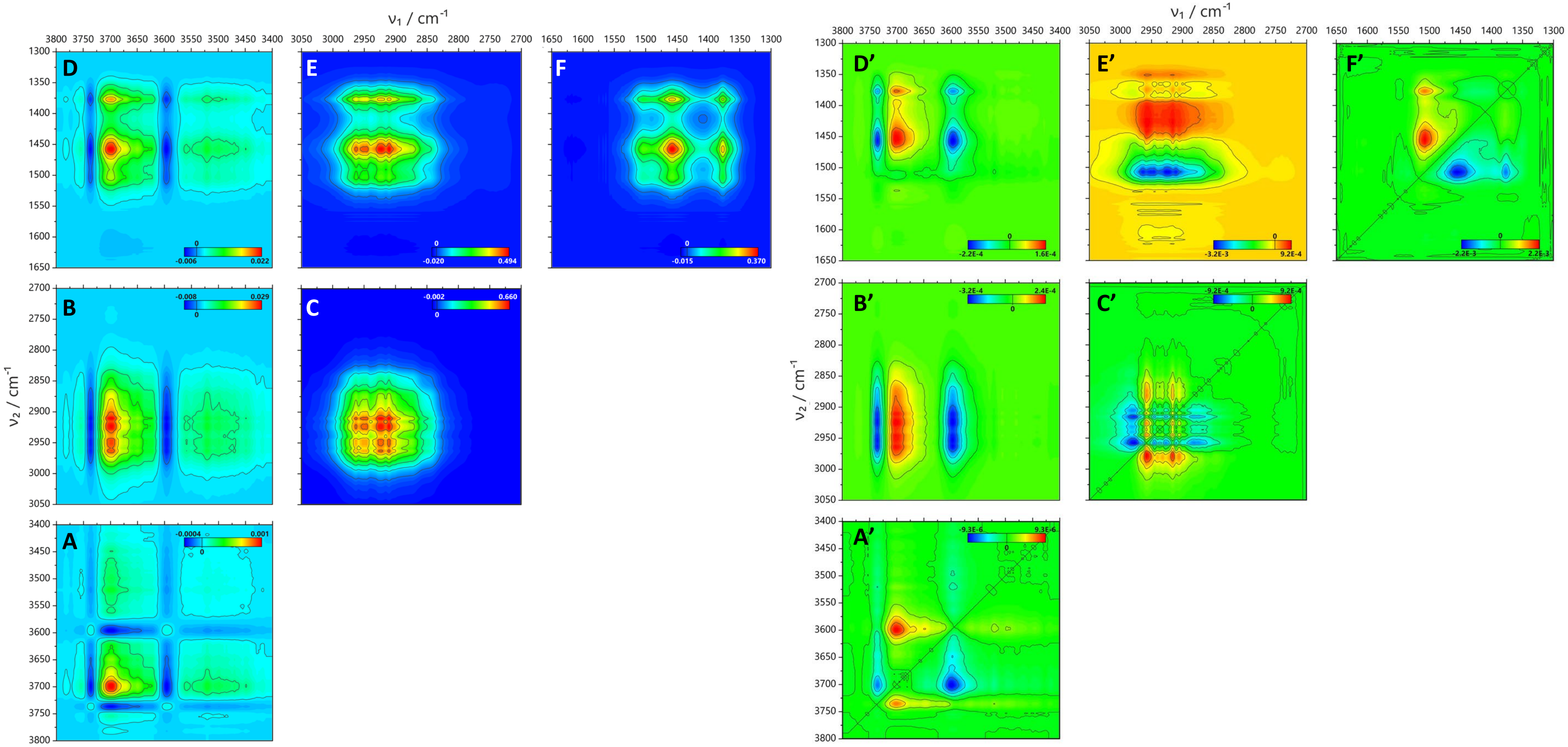

Fig. S5 2D CoS (synchronous – left, asynchronous – right) from operando FT-IR spectroscopic studies of PP cracking over micro-ZSM-5 zeolite.

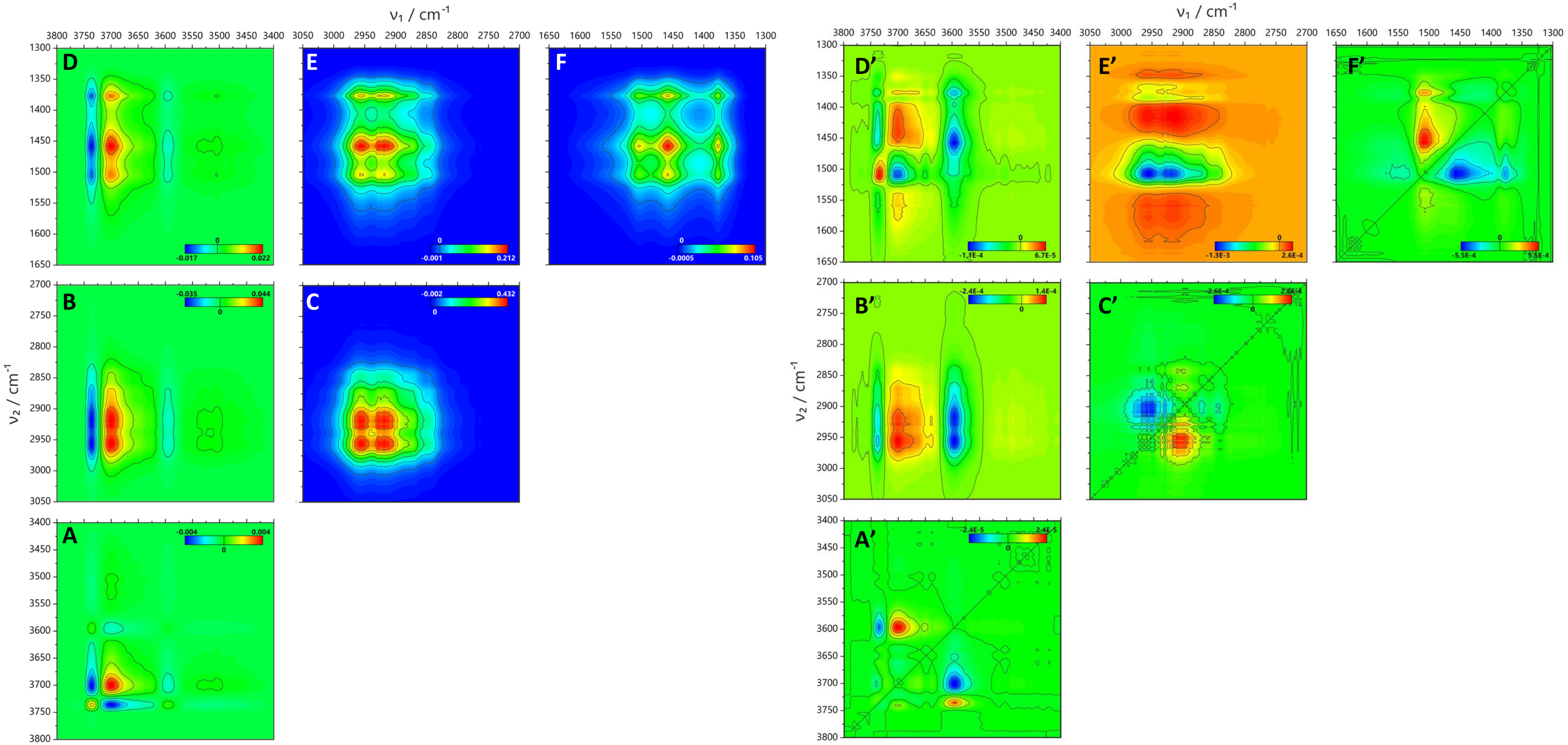

Fig. S6 2D CoS (synchronous – left, asynchronous – right) from operando FT-IR spectroscopic studies of PP cracking over deSi-ZSM-5 zeolite.

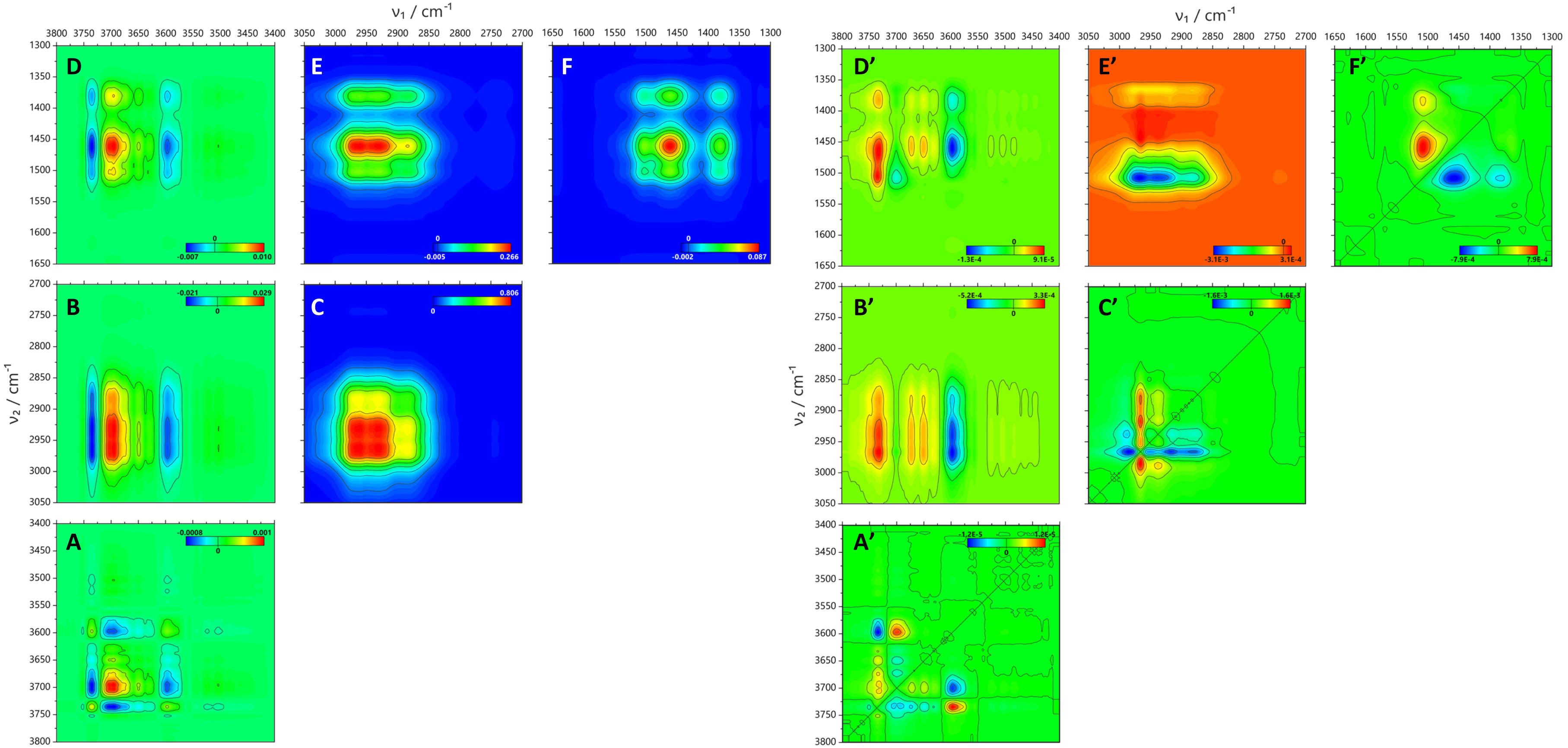

Fig. S7 2D CoS (synchronous – left, asynchronous – right) from operando FT-IR spectroscopic studies of PP cracking over deSi-ZSM-5&Ac zeolite.

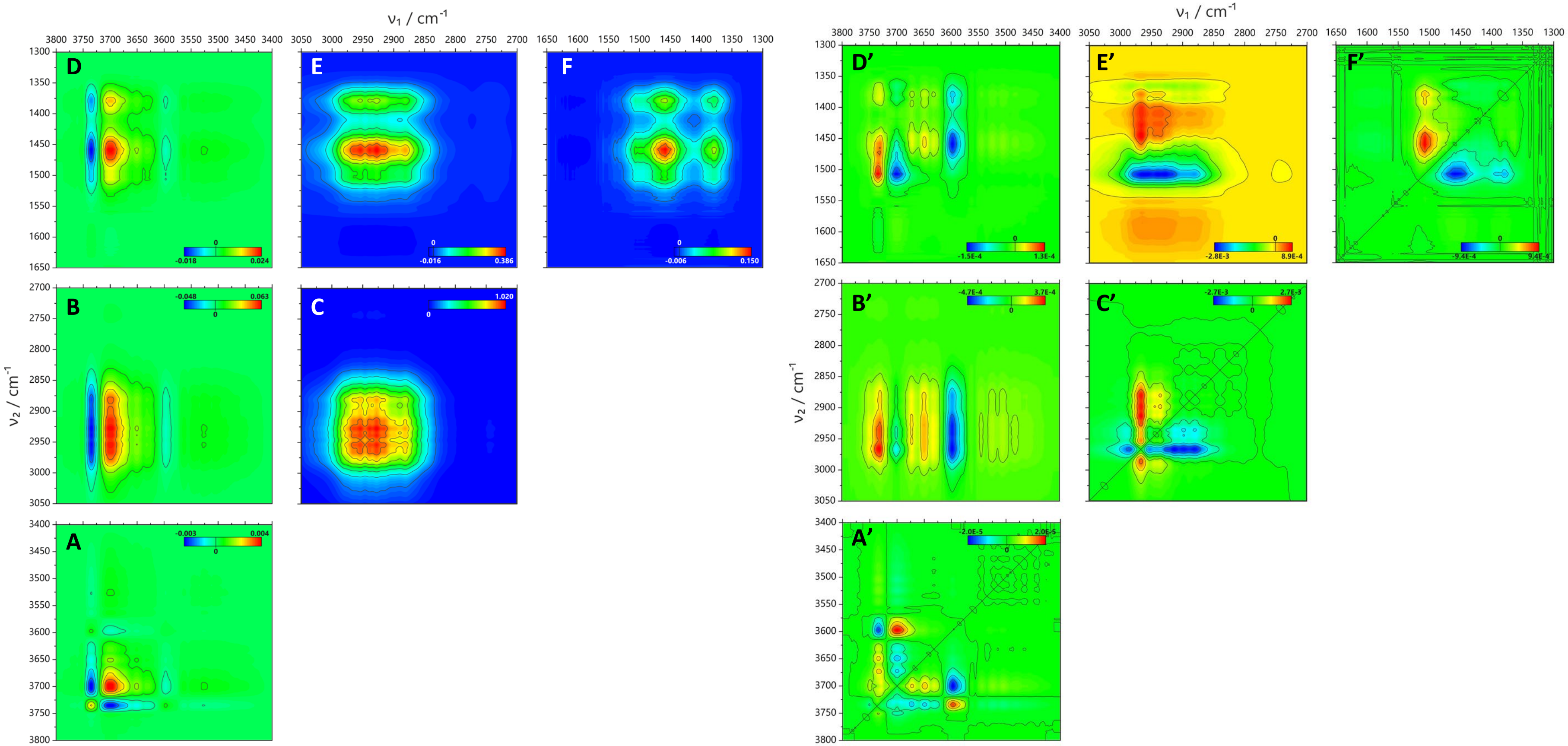

Fig. S8 2D CoS (synchronous – left, asynchronous – right) from operando FT-IR spectroscopic studies of PP cracking over deSi&PDA-ZSM-5 zeolite.

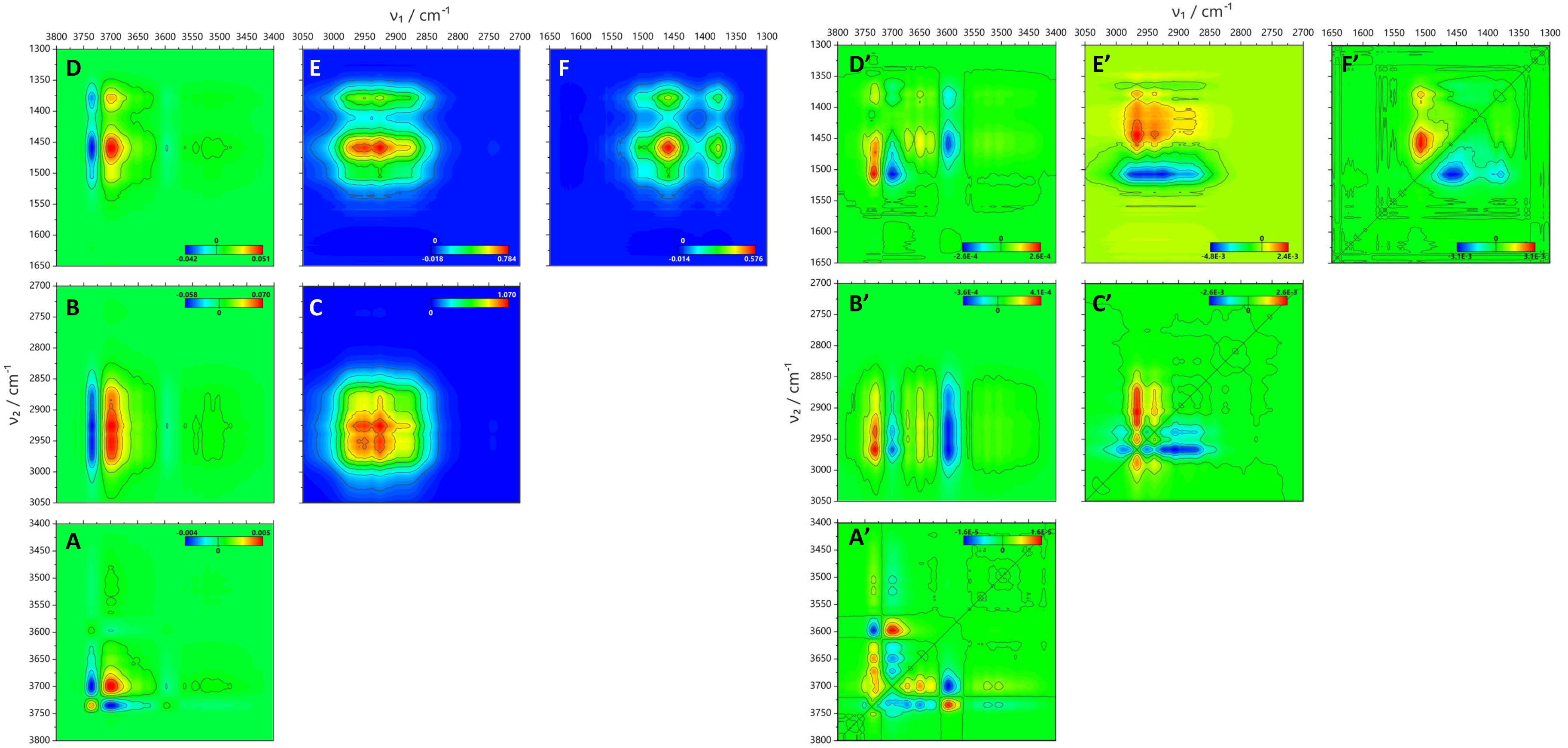

Fig. S9 2D CoS (synchronous – left, asynchronous – right) from operando FT-IR spectroscopic studies of PP cracking over deSi&PDA-ZSM-5&Ac zeolite.

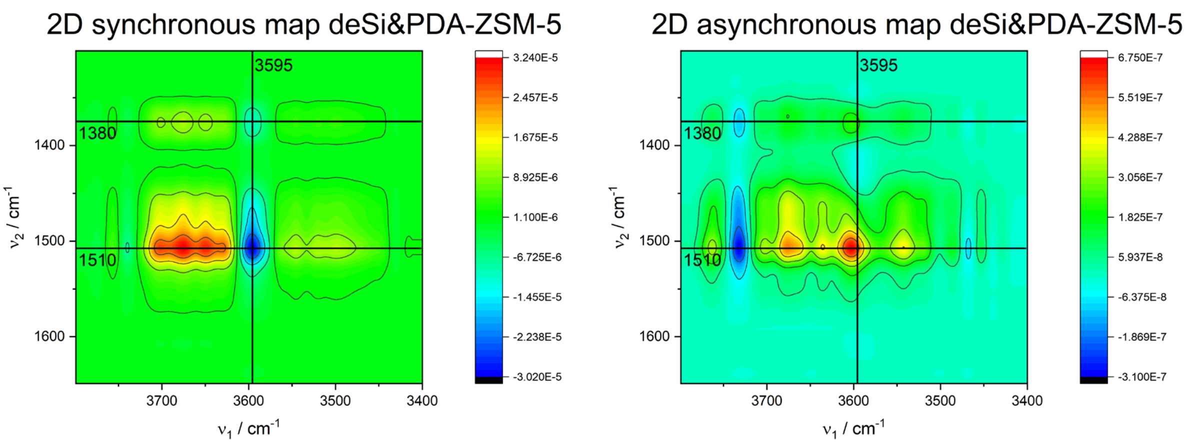

Fig. S10 Set of 2D CoS maps for sample deSi&PDA-ZSM-5 of spectra registered during cracking of PP after the period of initial cracking, i.e. after total realising of Si(OH)Al groups from water adsorption. It showed that the sign of correlation peak located at 3600 x 1510 cm^{-1} is different between the two maps; thus, the band changes at 1510 cm^{-1} precede the change of band 3610 cm^{-1}. The opposite was found at the initial stage of PP cracking.

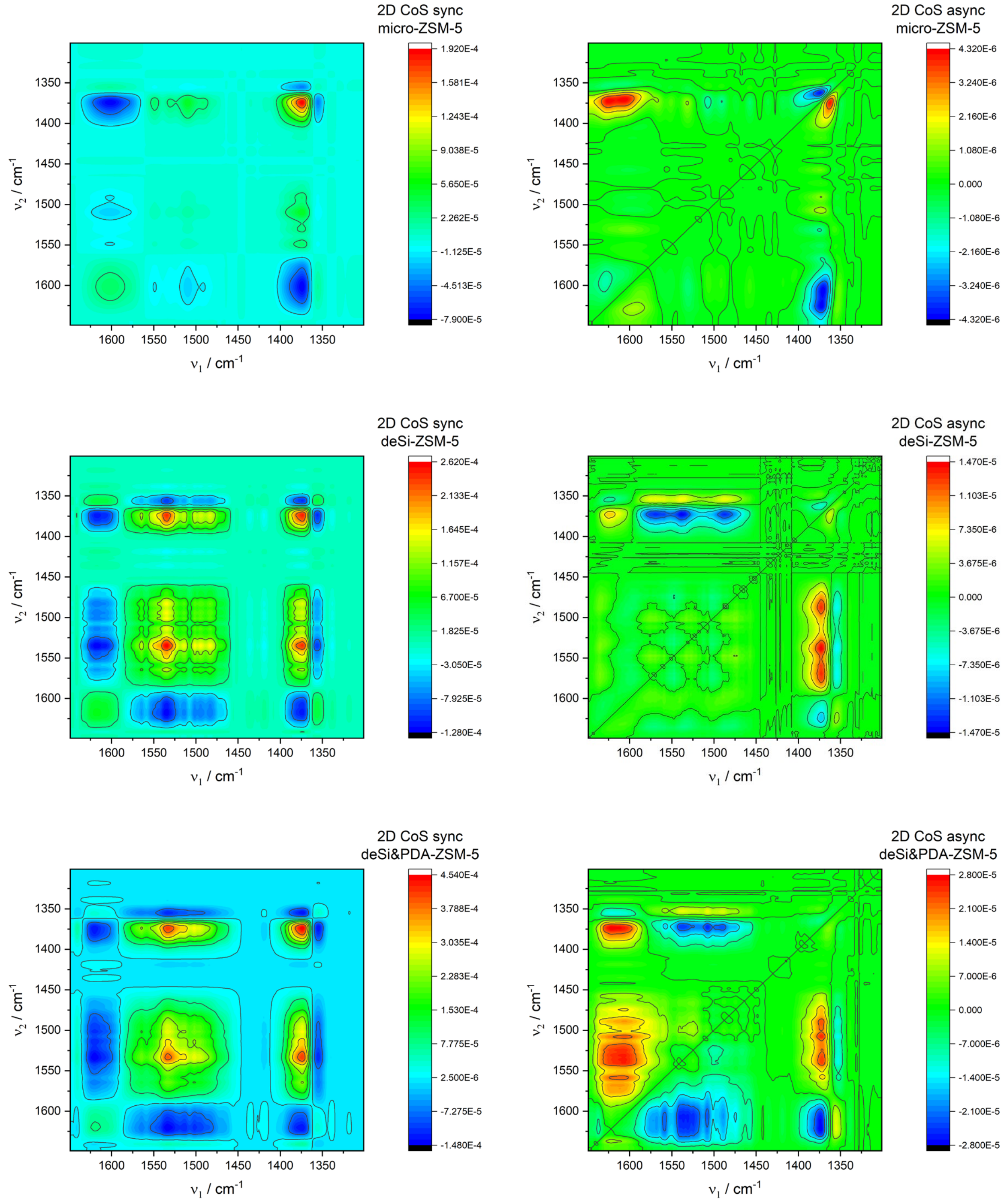

Fig. S11 Set of 2D CoS sync- and async-maps in region 1650 – 1300 cm^{-1} registered during coke burning off from studied samples.
